# Sizing Up Allometric Scaling Theory

**DOI:** 10.1371/journal.pcbi.1000171

**Published:** 2008-09-12

**Authors:** Van M. Savage, Eric J. Deeds, Walter Fontana

**Affiliations:** Department of Systems Biology, Harvard Medical School, Boston, Massachusetts, United States of America; University of Arizona, United States of America

## Abstract

Metabolic rate, heart rate, lifespan, and many other physiological properties vary with body mass in systematic and interrelated ways. Present empirical data suggest that these scaling relationships take the form of power laws with exponents that are simple multiples of one quarter. A compelling explanation of this observation was put forward a decade ago by West, Brown, and Enquist (WBE). Their framework elucidates the link between metabolic rate and body mass by focusing on the dynamics and structure of resource distribution networks—the cardiovascular system in the case of mammals. Within this framework the WBE model is based on eight assumptions from which it derives the well-known observed scaling exponent of 3/4. In this paper we clarify that this result only holds in the limit of infinite network size (body mass) and that the actual exponent predicted by the model depends on the sizes of the organisms being studied. Failure to clarify and to explore the nature of this approximation has led to debates about the WBE model that were at cross purposes. We compute analytical expressions for the finite-size corrections to the 3/4 exponent, resulting in a spectrum of scaling exponents as a function of absolute network size. When accounting for these corrections over a size range spanning the eight orders of magnitude observed in mammals, the WBE model predicts a scaling exponent of 0.81, seemingly at odds with data. We then proceed to study the sensitivity of the scaling exponent with respect to variations in several assumptions that underlie the WBE model, always in the context of finite-size corrections. Here too, the trends we derive from the model seem at odds with trends detectable in empirical data. Our work illustrates the utility of the WBE framework in reasoning about allometric scaling, while at the same time suggesting that the current canonical model may need amendments to bring its predictions fully in line with available datasets.

## Author Summary

The rate at which an organism produces energy to live increases with body mass to the 3/4 power. Ten years ago West, Brown, and Enquist posited that this empirical relationship arises from the structure and dynamics of resource distribution networks such as the cardiovascular system. Using assumptions that capture physical and biological constraints, they defined a vascular network model that predicts a 3/4 scaling exponent. In our paper we clarify that this model generates the 3/4 exponent only in the limit of infinitely large organisms. Our calculations indicate that in the finite-size version of the model metabolic rate and body mass are not related by a pure power law, which we show is consistent with available data. We also show that this causes the model to produce scaling exponents significantly larger than the observed 3/4. We investigate how changes in certain assumptions about network structure affect the scaling exponent, leading us to identify discrepancies between available data and the predictions of the finite-size model. This suggests that the model, the data, or both, need reassessment. The challenge lies in pinpointing the physiological and evolutionary factors that constrain the shape of networks driving metabolic scaling.

## Introduction

Whole-organism metabolic rate, *B*, scales with body mass, *M*, across species as [1]

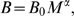
(1)where *B*_0_ is a normalization constant and *α* is the allometric scaling exponent, typically measured to be very close to 3/4 [2]. The empirical regularity expressed in Equation 1 with *α* = 3/4 is known as Kleiber's Law [3],[4].

Many other biological rates and times scale with simple multiples of 1/4. For example, cellular or mass-specific metabolic rates, heart and respiratory rates, and ontogenetic growth rates scale as *M* ^−1/4^, whereas blood circulation time, development time, and lifespan scale close to *M* ^1/4^ [5]–[9]. Quarter-power scaling is also observed in ecology (e.g., population growth rates) and evolution (e.g., mutation rates) [2],[10],[11]. The occurrence of quarter-power scaling at such diverse levels of biological organization suggests that all these rates are closely linked. Metabolic rate seems to be the most fundamental because it is the rate at which energy and materials are taken up from the environment, transformed in biochemical reactions, and allocated to maintenance, growth, and reproduction.

In a series of papers starting in 1997, West, Brown, and Enquist (WBE) published a model to account for the 3/4-power scaling of metabolic rate with body mass across species [1], [12]–[14]. The broad theory of biological allometry developed by WBE and collaborators attributes such quarter-power scaling to near-optimal fractal-like designs of resource distribution networks and exchange surfaces. There is some evidence that such designs are realized at molecular, organelle, cellular, and organismal levels for a wide variety of plants and animals [2],[14].

Intensifying controversy has surrounded the WBE model since its original publication, even extending to a debate about the quality and analysis of the data [15]–[28]. One of the most frequently raised objections is that the WBE model cannot predict scaling exponents for metabolic rate that deviate from 3/4 [16],[29], even though the potential for such deviations was appreciated by WBE themselves [1]. If this criticism were true, WBE could not in principle explain data for taxa whose scaling exponents have been reported to be above or below 3/4 [29]–[34], or deviations from 3/4 that have been observed for small mammals [35]. Likewise, the WBE model would be unable to account for the scaling of maximal metabolic rate with body mass, which appears to have an exponent of 0.88 [36]. It is important to note that the actual nature of maximal metabolic rate scaling is, however, not without its own controversy; see [37] for an argument that maximal metabolic rate scales closer to 3/4 when body temperature is taken into consideration.

Much of the work aimed at answering these criticisms has relied on alteration of the WBE model itself. Enquist and collaborators account for different scaling exponents among taxonomic groups by emphasizing differences in the normalization constant *B*_0_ of Equation 1 and deviations from the WBE assumptions regarding network geometry [26], [38]–[40]. While these results are suggestive, it remains unclear whether or not WBE can predict exponents significantly different from 3/4 and measurable deviations from a pure power law even in the absence of any variation in *B*_0_ and with networks following exactly the geometry required by the theory. Although WBE has been frequently tested and applied [40]–[53], it is remarkable that no theoretical work has been published that provides more detailed predictions from the original theory. Also, work aimed at extending WBE by relaxing or modifying some of its assumptions has hardly been complete; many variations in network structure might have important and far-reaching consequences once properly analyzed. This is what we set out to do in the present contribution. We show that a misunderstanding of the original model has led to the claim that WBE can only predict a 3/4 exponent. This is because many of the predictions and tests of the original model are derived from leading-order approximations. In this paper we derive more precise predictions and tests.

For the purpose of stating our conclusions succinctly, we refer to the “WBE framework” as an approach to explaining allometric scaling phenomena in terms of resource distribution networks (such as the vascular system) and to the “WBE model” as an instance of the WBE framework that employs particular parameters specifying geometry and (hydro)dynamics of these networks [1],[14]. (We shall detail these assumptions and define terminology more accurately in section “Assumptions of the WBE model”.)

Our main findings are: 1. The 3/4 exponent only holds exactly in the limit of organisms of infinite size. 2. For finite-sized organisms we show that the WBE model does not predict a pure power-law but rather a curvilinear relationship between the logarithm of metabolic rate and the logarithm of body mass. 3. Although WBE recognized that finite size effects would produce deviations from pure 3/4 power scaling for small mammals and that the infinite size limit constitutes an idealization [1], the magnitude and importance of finite-size effects were unclear. We show that, when emulating current practice by calculating the scaling exponent of a straight line regressed on this curvilinear relationship over the entire range of body masses, the exponent predicted by the WBE model can differ significantly from 3/4 without any modifications to its assumptions or framework. 4. When realistic parameter values are employed to construct the network, we find that the exponent resulting from finite-size corrections comes in at 0.81, significantly higher than the 3/4 figure based on current data analysis. 5. Our data analysis indeed detects a curvilinearity in the relationship between the logarithm of metabolic rate and the logarithm of body mass. However, that curvilinearity is opposite to what we observe in the WBE model. This implies that the WBE model needs amendment and/or the data analysis needs reassessment.

Beyond finite-size corrections we examine the original assumptions of WBE in two ways. First, we vary the predicted switch-over point above which the vascular network architecture preserves the total cross-sectional area of vessels at branchings and below which it increases the total cross-sectional area at branchings. These two regimes translate into different ratios of daughter to parent radii at vessel branch points. Second, we allow network branching ratios (i.e., the number of daughter vessels branching off a parent vessel) to differ for large and small vessels. We analyze the sensitivity of the scaling exponent with respect to each of these changes in the context of networks of finite size. This approach is similar in spirit to Price et al. [40], who relaxed network geometry and other assumptions of WBE in the context of plants. In the supplementary online material Text S1, we also argue that data analysis should account for the log-normal distribution of body mass abundance, thus correcting for the fact that there are more small mammals than large ones. Despite differences in the structure and hydrodynamics of the vascular systems of plants and animals [1],[13], detailed models of each yield a scaling exponent of 3/4 to leading-order. In the present paper, we focus on the WBE model of the cardiovascular system of mammals. All of our assumptions, derivations, and calculations should be interpreted within that context. Finite-size corrections and departures from the basic WBE assumptions are important in the context of plants as well, as shown in recent studies by Enquist and collaborators [26], [38]–[40].

In final analysis, we are led to the seemingly incongruent conclusions that (1) many of the critiques of the WBE framework are misguided and (2) the exact (i.e., finite-size corrected) predictions of the WBE model are not fully supported by empirical data. The former means that the WBE framework remains, once properly understood, a powerful perspective for elucidating allometric scaling principles. The latter means that the WBE model must become more respectful of biological detail whereupon it may yield predictions that more closely match empirical data. Our work explores how such details can be added to the model and what effects they can have.

The paper is organized as follows. For the sake of a self-contained presentation, we start with a systematic overview of the assumptions, both explicit and implicit, underlying the WBE theory (section “Assumptions of the WBE model”). In Text S1, we provide a detailed exposition of the hydrodynamic derivations that the model rests upon. These calculations are not original, but they have not appeared to a full extent before in the literature. While nothing in section “Assumptions of the WBE model” is novel, there seems to be no single “go to” place in the WBE literature that lays out all components of the WBE theory. Our paper then proceeds with a brief derivation of the exact, rather than approximate, relationship between metabolic rate and body mass (section “Derivation of the 3/4 scaling exponent”). We then calculate the exact predictions for scaling exponents for networks of finite size (section “Finite-size corrections to 3/4 allometric scaling”) and revisit certain assumptions of the theory (section “Making the WBE model more biologically realistic”). In section “Comparison to empirical data” we compare our results to trends detectable in empirical data. We put forward our conclusions in the Discussion section.

## Model

### Assumptions of the WBE Model

The WBE model rests on eight assumptions. Some of these assumptions posit the homogeneity of certain parameters throughout the resource distribution network. Any actual instance of such a network in a particular organism will presumably exhibit some heterogeneity in these parameters. The object of the theory is a network whose parameters are considered to be averages over the variation that might occur in any given biological instance. For the sake of brevity, we refer to such a network as an “averaged network”. The impact of parameter heterogenity on the scaling exponent is very difficult to determine analytically. (Section “Changing branching ratio across levels” addresses a modest version of this issue numerically.)

#### Assumption 1. The distribution network determines the scaling relationship

The relationship between metabolic rate and body mass is dominated by the structure and dynamics of the resource distribution network, which for most animals is the cardiovascular system. This assumption constitutes the core of the WBE framework. The vascular system is directly tied to metabolic rate, because the flow dynamics through the network and the number of terminal points (capillaries) constrain the rates at which cells and tissues are supplied with oxygen and nutrients needed for maintenance. At the same time, the vascular system is directly tied to body volume (and thus body mass), because network extent and structure must be such that its terminal points can service (and thus cover) the entire body volume. It follows that the relationship between metabolic rate and body mass must be constrained - and WBE assume it is dominated - by the structural and flow properties of the cardiovascular system. It should be noted that Assumption 1 could be true even if other assumptions of WBE are false. (For a recent example with plant architecture and data, see Price et al. [40].) In other words, even if the cardiovascular system does not drive the particular allometry between mass and metabolic rate, the cardiovascular system must be *consistent* with the observed scaling.

#### Assumption 2. The distribution network is hierarchical

**Figure 1:**
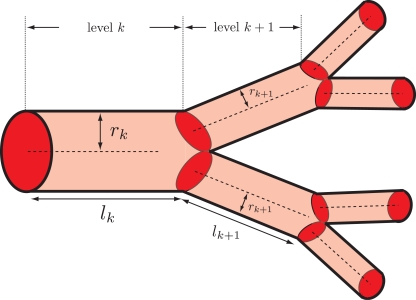
Schematic vessel architecture and branching. A vessel at level *k* branches into two daughter vessels at level *k*+1. The branching ratio is thus *n* = 2. The radii, *r_k_*_+1_, and lengths, *l_k_*_+1_, of the two daughter vessels are identical by Assumption 3. The ratios of the radii and lengths at level *k*+1 to those at level *k* are defined as *γ*, *β*_>_ and *β*_<_ in Equations 2 and 3. The choice of *β*_>_ for the radial ratio corresponds to area-preserving branching and of *β*_<_ to area-increasing branching. In the WBE model, the cardiovascular system is composed of successive generations of these vascular branchings, from level 0 (the heart) to level *N* (the capillaries).

To say that the cardiovascular system is hierarchical amounts to assuming that there is a consistent scheme for labeling different levels of vasculature (Figure 1), proceeding from the heart (level 0) to the capillaries (level *N*). This assumption is not exactly true. For example, the number of levels from the heart to the capillaries in the coronary artery is smaller than the number of levels from the heart to the capillaries in the foot [54]. Yet, the hierarchical structure is evident in images of whole-body vasculature, and is posited to constitute a good approximation for analyzing properties of an averaged network.

#### Assumption 3. Vessels within the same level of the hierarchy are equivalent

All the vessels at the same level of the network hierarchy have the same radius, length, and flow rate. Again, this assumption is not strictly true but provides a tractable way to study an averaged network.

#### Assumption 4. The branching ratio is constant

The number of daughter vessels at a branching junction—the branching ratio *n*—is assumed to be constant both within and across levels. By definition of the branching ratio, the total number of vessels within level *k* is *N_k_* = *n^k^*. The total number of vessels in the previous level is *N_k_*_−1_ = *n^k^* ^−1^, thus *n* = *N_k_*/*N_k_*_−1_. The constancy of *n* provides a good approximation for describing the properties of an averaged network. We will show in section “Derivation of the 3/4 scaling exponent” that the value of *n* does not affect the leading-order scaling (infinite-size limit) of the allometry. However, it does slightly affect the corrections to 3/4 for organisms of finite size. In the original WBE paper, a constant branching ratio *n* is listed as a consequence of Assumption 6 (below), which uses a Lagrange multiplier calculation to minimize the energy required for fluid flow through the vascular hierarchy. That claim is incorrect because there are not enough Lagrange constraints to determine this additional ratio. Although the constancy of the branching ratio plays a pivotal role in relating vessel radii and lengths at one level to those at the subsequent level, deviations from Assumptions 3 and 4 were not believed to have much effect on the predicted scaling exponent based on numerical work on side branchings by Turcotte [55].

#### Assumption 5. The network is space filling

Resource distribution networks are space-filling in the sense that they must feed (though not necessarily touch) every cell in the body. This assumption determines how vessel lengths at one level relate to vessel lengths at the next level. Although this assumption seems simple and intuitively appealing, it has a precise meaning that is not easily conveyed by this terminology. A single capillary feeds a group of cells, which constitute the service volume, *v_N_* (*N* denotes the terminal branching level), of a capillary. Since all living tissue must be fed, the sum over all these service volumes must equal the total volume of living tissue, *V*_tot_ = *V*_cap_ *v_N_*, where *N*_cap_ is the number of capillaries, that is, the number of vessels at the terminal level *N*, *N_N_* = *N*_cap_. This argument can be repeated for vessels one level above the capillaries (level *N*−1), only now each of those vessels must service a group of capillaries that comprises some volume, *v_N_*_−1_. Again, the sum over all these *N_N_*_−1_ service volumes must equal the total volume of living tissue, *V*_tot_ = *N_N_*_−1_ *v_N_*_−1_, because that is the volume the capillaries must maintain. Iterating this argument over all network levels yields *N_N_v_N_* = *N_N_*_−1_ *v_N_*_−1_ = … = *N*_0_ *v*_0_. This is the meaning of space filling. If a vessel at level *k* of the hierarchy has length *l_k_* (see Figure 1), we can think of the service volumes as varying with 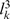 (e.g., a sphere with diameter *l_k_* or cube with length *l_k_*). Thus, 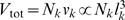. Since *V*_tot_, the total volume of living tissue, is independent of *k*, we obtain the scale-free ratio,
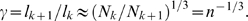
(2)WBE assume this relation to be valid throughout the network, although it becomes less realistic for small values of *k*, i.e. near the heart [1]. (The notation of the model is not optimal. For example, *N* with a subscript denotes the number of vessels at the level indicated by the subscript, but *N* without a subscript denotes the level of the capillaries. We refrain, however, from redefining established notation.)

#### Assumption 6. The energy loss of fluid flow through the network is minimized

The work done to pump blood from the heart to the capillaries has been minimized by natural selection. This assumption relates the radius of vessels at one level of the network to the radius of vessels at the next level. The trial-and-error feedback implicit in evolutionary adaptation and development has led to transport networks that, on average, minimize the energy required for flow through the system. There are two independent contributions to energy loss: energy dissipated by viscous forces and energy loss due to pulse reflection at branch points. Dissipation is the major cause of energy loss in flow through smaller vessels, such as capillaries and arterioles, because a high surface-to-volume ratio subjects a larger fraction of the blood volume to friction from vessel walls. Energy loss to wave reflections, on the other hand, has the potential to be dominant in larger vessels, such as arteries, where flow is pulsatile.

Reflection can be entirely eliminated by equalizing the opposition to fluid flow before and after the branching of a vessel [54],[56]. This opposition is called impedance, and the equalization is referred to as impedance matching. Energy loss to dissipation, however, can be minimized but never eliminated by network architecture because friction always exists between blood and the vessel walls. A detailed calculation of these energy losses involves an analysis of fluid flow in elastic tubes using the Navier-Stokes and the Navier equations, Lagrange multiplier methods, and other techniques. We provide a roadmap for these calculations in Text S1 (see also Figure S1, Figure S2, and Figure S3). The main result of the analysis is twofold. First, minimizing dissipation leads to a cube-law [57] for the radii, *r_k_*, at branch points,
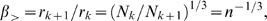
(3)which indicates that the total cross-sectional area will *increase* at branch points and thus, by continuity, will result in a slowing of the blood flow rate. Second, when vessels are large, minimizing reflections leads to a square-law for radii,
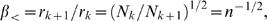
(4)which *preserves* the cross-sectional area at branch points and results in a constant blood flow rate across the branching. When vessels are small, however, minimizing reflections also leads to a cube-law analogous to the relationship in (3). We refer to the two different forms of radius scaling (*β*_>_ and *β*_<_) as “area-increasing” and “area-preserving” branching, respectively.

The ratio of radii in a real system probably changes continuously throughout the network. It seems, however, a reasonable approximation to assume that the ratios (3) and (4) dominate two regions, and that within each region the network is self-similar, meaning that the branching ratio *n* is constant. Empirical data provide some support for the existence of these two regions [54], but it is quite difficult to determine the transition between them, either empirically or theoretically. WBE argue that the level at which the transition occurs, *k̅* (as counted from the heart, level 0), is always a *fixed* number of levels *N̅* away from the capillaries (level *N*): *N̅* = *N*−*k̅* = const. This means that the transition depends only on the vessel radii, which partially dictate the resistance to flow, and thus, that the transition always occurs at a fixed radius. Notice, however, that a switch-over from area-preserving to area-increasing branching does not necessarily coincide with a switch-over from impedance matching (for pulsatile flow) to minimization of dissipation (for viscous flow), because impedance matching alone already implies a transition from area-preserving to area-increasing branching (see Text S1). Finally, and rather importantly, Assumption 6 is used to prove that total blood volume, *V*_blood_, is directly proportional to body mass *M* as explained in Text S1.

#### Assumption 7. Capillary characteristics are the same across species

Flow rate, length, radius, hematocrit, and all other structural and physiological traits of capillaries are independent of body size. It is this assumption that allows comparisons among organisms. All previous assumptions specify the structure of the vascular system *within* an organism, but Assumption 7 sets the scale *across* organisms. WBE view capillaries as fundamental building blocks of the system that remain constant as organisms are scaled up in size. Indeed, a large amount of empirical data for mammals shows no systematic trend of capillary size or red blood cell size with body size [9],[58].

#### Assumption 8. Capillaries are the only exchange surfaces and thus directly relate blood flow rate to oxygen supply in tissues

All transfer of resources happens through the terminal exchange surfaces, i.e., at the level of capillaries and not at other levels in the network. With regard to oxygen, this assumption is well founded because capillary size and structure are likely to have been under selection pressure to facilitate the release of oxygen by red blood cells and hemoglobin [59]–[62]. This is presumably why red blood cell diameter closely matches capillary diameter [9], which facilitates conversion from oxygen supply into metabolic rate [63],[64]. Indeed, increased blood flow rate signals increased oxygen supply and metabolic rate—a fact exploited in neuroimaging techniques, such as Blood Oxygenation Level Dependent (BOLD) fMRI [65],[66].

As mentioned in the introduction, it is useful to clarify some terminology that we will employ in this work. Throughout, we refer to “the WBE model” as any version based on Assumptions 1–8 above. This includes the original infinite-size limit as well as the finite-size version whose analysis we carry out in this paper. We refer to the “canonical WBE model” when singling out the predictions of the WBE model obtained with the original parameter values, such as *N̅* = 24 and *n* = 2. Finally, we distinguish between the WBE model and the “WBE framework”. The latter is a stance that seeks to explain allometric scaling in terms of the physical structure and dynamics of resource distribution networks without necessarily conforming to Assumptions 2–8. It is important to note that, while problems might exist with any given model within this framework, those problems do not invalidate the importance of considering resource distribution networks to understand allometric scaling phenomena.

### Derivation of the 3/4 Scaling Exponent

Using the above assumptions, we can derive how metabolic rate, *B*, varies with body mass, *M*, which is the fundamental result of WBE. The key insight is that body mass is proportional to blood volume (following from Assumption 6) and that blood volume is the sum of the volumes of the vessels over all the levels of the network. Using Assumptions 2–6, this sum can be expressed in terms of properties of capillaries, providing a direct link to metabolic rate (owing to Assumption 8). Upon expressing blood volume in terms of capillary properties, we can separate terms of the sum that are invariant (by Assumption 7) from others that vary with the total number of capillaries. The total number of capillaries is directly proportional to the whole-oganism metabolic rate, because each capillary supplies resources at the same rate regardless of organism size (Assumption 7). This ties body mass to metabolic rate. We now provide the formal derivation.

Using Assumptions 2–4 the total blood volume or total network volume (assuming the network is completely filled with blood and ignoring the factor of 2 that may arise from blood in the venous system, which returns blood to the heart) can be expressed as the sum
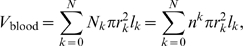
(5)where the volume of a vessel is that of a cylinder. Next, we use the scale-free ratios *γ*, *β*_<_, and *β*_>_, defined by Equations 2–4 resulting from Assumptions 5 and 6, to connect level *k* to successively higher levels and all the way to the capillary level *N*:
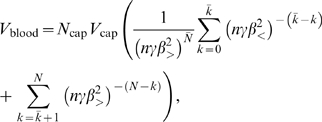
(6)where 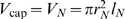 is the volume of a capillary and *N*_cap_ = *N_N_* = *n^N^* the number of capillaries. The first sum ranges over the area-preserving regime and the second sum is over the area-increasing regime. The first sum is a standard geometric series. Observing that 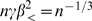 then yields
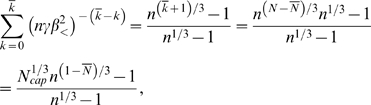
(7)where *N̅* is the fixed number of levels from the capillaries to the level where the transition from area-increasing to area-preserving branching occurs. Since 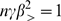, we have 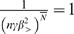, and the second sum in Equation 6 is simply
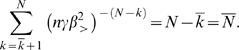
(8)Combining these results we have
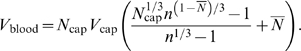
(9)This equation can be re-expressed as
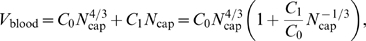
(10)where
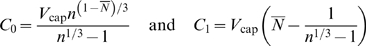
(11)are both constant with respect to body mass. Equation 10 will play a fundamental role in the following sections.

Given this simple relation between total blood volume (or network volume) and the number of capillaries, it is straightforward to relate metabolic rate, *B*, to body mass, *M*. Using Assumption 8, the whole-body metabolic rate is just the sum total of the metabolic rates enabled by the resources delivered through each capillary. Let the contribution to total metabolic rate enabled by a capillary be *B*_cap_. By Assumption 7 *B*_cap_ is constant across organisms. Thus, *B* = *N*_cap_ *B*_cap_, or simply *B*∝*N*_cap_. Inserting this into Equation 10, invoking Assumption 7 that *V*_cap_ is independent of body mass, and using Assumption 6 to recognize that *M*∝*V*_blood_ yields
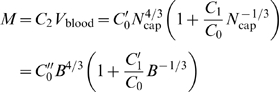
(12)with *C*_2_ a constant, 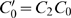, 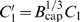, and 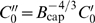 are new constants.

Letting the number of levels in the cardiovascular system, *N*, tend to infinity—which necessarily means that body mass, *M*, and metabolic rate, *B*∝*N*_cap_ = *n^N^*, become infinitely large—we conclude that
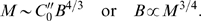
(13)This is the celebrated result that has been empirically observed for nearly a century. Equation 13 is approximately true as long as
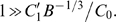
(14)


It is essential to realize that the prediction of a 3/4 scaling relationship *only* holds in the infinite *M*-limit. The approximation becomes less accurate as organisms become smaller, corresponding to smaller metabolic rate *B*. The exact relationship is Equation 12, or 10, which does not predict a pure power law but a curvilinear graph of ln *B* versus ln *M*. Forcing such a curve to fit a straight line will therefore not produce an exact value of 3/4, except when the magnitude of the correction term is small compared with 1 (see Equation 14), the measurement error, or the residual variation in the empirical data. Given that the importance of these deviations will be larger for smaller organisms, it would in principle be interesting to look more carefully at finite-size effects for small fish or plants, because the smallest mammals are considerably larger than the smallest fish or plants [25],[26],[29],[33], although we do not perform such an analysis here. Different taxa often span different ranges of body size and exhibit a particular relative proportion of small to large organisms. These characteristics will likely lead to different measured scaling exponents.

We conclude that the WBE model actually predicts variation in scaling exponents due to finite-size terms whose magnitude depends on the absolute range of body masses for a given taxonomic group. These predictions can be tested against the allometric exponents reported in the empirical literature.

## Results

### Finite-Size Corrections to 3/4 Allometric Scaling

To quantify finite-size corrections, we focus on Equation 10 because the blood volume, *V*_blood_ (∝*M*), and the number of capillaries, *N*_cap_ (∝*B*), are really the fundamental parameters of the theory. Proceeding in this way, we avoid the additional constants *C*_2_ and *B*_cap_. By inspecting Equation 10, we see that finite-size effects can become manifest in two different ways. First, even in the absence of network regions with area-increasing branching (*N̅* = 0), there are corrections to the 3/4 scaling exponent. Second, the switch-over point *N̅* from area-preserving branching to area-increasing branching determines the relative contributions of these two network regimes, and has the potential to considerably influence the scaling exponent. To quantify these effects, we consider three cases: (i) a network with only area-preserving branching (section “Networks with only area-preserving branching”), (ii) a network with only area-increasing branching (section “Networks with only area-increasing branching”), and (iii) a mixture of the two with a transition level (section “Networks with a transition from area-preserving to area-increasing branching”).

#### Networks with only area-preserving branching

**Figure 2:**
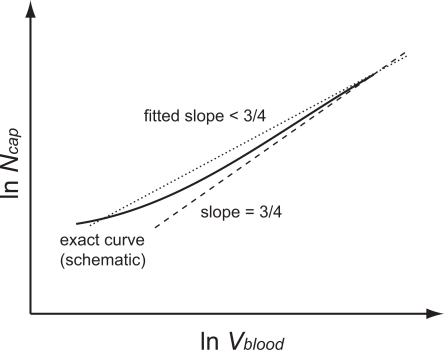
Schematic scaling relation for finite-size corrections in networks with only area-preserving branching. The dashed line schematically depicts the 3/4 power law that relates the number of capillaries, *N*_cap_, to the blood volume *V*_blood_. This scaling relationship is a straight line in logarithmic space (ln *N*_cap_ versus ln *V*_blood_) and represents the leading-order behavior in the limit of infinite blood volume and organism size. The solid line dramatizes the curvature for the scaling relation for finite-size networks obtained when vessel radii are determined solely by area-preserving branching. The dotted line illustrates the consequences of a linear regression on the curve for finite-size organisms (solid line). Since the solid line depicts the predicted curvilinear relationship that deviates above and away from the infinite-size asymptote, Equation 16, the WBE model predicts that fits to data for organisms whose vascular networks are built only with area-preserving branching will yield scaling exponents smaller than 3/4.

A network in which all levels are area-preserving corresponds to a switch-over point at *k̅* = *N*, so *N̅* = 0. Equation (10) still holds, and we have 
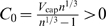
 and 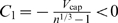, with the simple ratio |*C*_1_|/|*C*_0_| = *n* ^−1/3^. To understand how this affects a log-log plot of V_blood_ versus the number of capillaries *N*_cap_ (see Figure 2) and thus the overall scaling exponent, we express Equation 10 in the form
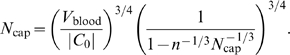
(15)In the limit *N*_cap_→∞ we obtain a scaling exponent of 3/4. However, as *N*_cap_ decreases, the second factor in Equation 15 increases, resulting in values of *N*_cap_ on the left of (15) that are larger than values in the case of a pure 3/4 power-law. A log-log plot of this curve will asymptote to a straight line with a slope of 3/4 for large *N*_cap_ and bend up and away from it as *N*_cap_ decreases. Regressing a straight line on this curve will yield a scaling exponent below 3/4, as shown schematically in Figure 2.

We can make this quantitative by implicit differentiation, which yields the tangent to the curve defined by Equation 15 as
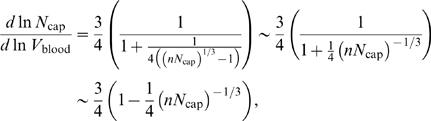
(16)where the last expression is the leading-order correction for large *N*_cap_. Indeed, from Equation 16 we see that the tangent to the curve becomes shallower as *N*_cap_ decreases.

In order to more directly compare these finite-size effects with empirical data, we need to develop an approach that mirrors the absolute size and size range of real taxonomic groups and organisms. To do so, we imagine constructing a group of organisms of differing sizes; the smallest organism in this group corresponds to the smallest network and the largest organism corresponds to the largest network. Each organism possesses a network with a specific value of *N*_cap_ and *V*_blood_ determined by Equation 10. The scaling exponent for such a group would correspond to the slope obtained from a linear regression of ln *N*_cap_ on ln *V*_blood_ for all of the data points obtained for all of the organisms in that group. The influence of absolute size on the scaling exponent can then be captured by fixing the size range covered by a group (e.g., 8 orders of magnitude for mammals) and measuring the change in the exponent that results from increasing the size of the organisms in the group. Consequently, if a group has a size range from the smallest to the largest organism that spans 26 levels of the vascular system, then we would compare the exponent obtained for a group covering *N* = 4 to *N* = 30 levels to that obtained for a group covering *N* = 24 to *N* = 50 levels.

We now use this approach for both analytical approximations and numerical calculations. First, we can estimate analytically the exponent that would be measured for a group of organisms spanning a range of levels and thus a range of body masses. If a power-law represents a good fit for a group, we can approximate its slope using only the network (blood) volume and capillary number for the smallest and largest organisms. Hence, the slope of the regression line could be estimated by calculating the total change in ln *N*_cap_ across the group and dividing it by the total change in ln *V*_blood_ across the group. Using Equation 15 along with standard expansions and approximation methods, we find the leading-order terms in the limit of large *N*_cap_ to be
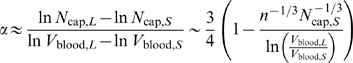
(17)wherein we used the fact that *N*_cap,*S*_≪*N*_cap,*L*_. The subscripts *S* and *L* denote the smallest and largest organism in a group, respectively. We can further simplify this expression by recalling that body sizes of mammals range over eight orders of magnitude in going from a shrew to a whale, hence ln(*V*_blood,*L*_/*V*_blood,*S*_)≈8 ln 10. Note, however, that the numerator in Equation 17 depends on *N*_cap,*S*_, thus capturing absolute size effects, not just the range under consideration.

**Figure 3:**
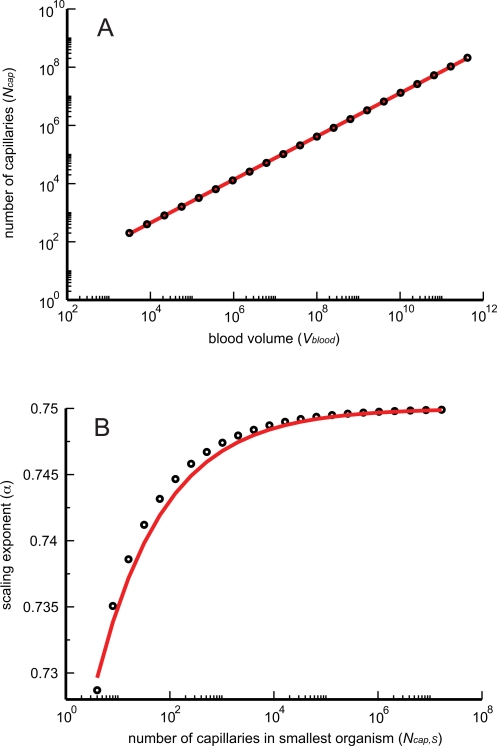
Finite-size corrections for networks with only area-preserving branching. (A) The logarithm of the number of capillaries is regressed with ordinary least squares (OLS) on the logarithm of blood volume for a set of artificial networks, spanning 8 orders of magnitude, built with only area-preserving branching. In this particular example the scaling exponent is determined to be 0.743, very close to 3/4. Black circles: numerical values. Red curve: power-law regression. (B) A scaling exponent *α* is determined by OLS regression for each group of artificial networks spanning roughly 8 orders of magnitude in body mass (blood volume). Exponents so-determined are paired with the size of the smallest network (as measured by the number of capillaries, *N*_cap,*S*_) in the corresponding group. Groups are built by systematically increasing the size of the smallest network, while always maintaining a range of 8 orders of magnitude in body volume (mass), resulting in the depicted graph. In all cases the branching ratio was *n* = 2. Black circles: numerical values. Red curve: analytical approximation, Equation 17.

To test our derivations, we numerically computed and analyzed data that were generated in accordance with WBE assumptions. We start by constructing a group of different “model organisms”, each consisting of a distinct number of levels, thereby yielding a particular *N*_cap_ and *V*_blood_. One might think of such a group as comprising organisms belonging to the same taxon. A group might include, for example, a smallest organism with 8 levels and a largest organism with 30 levels of vascular hierarchy. We ensured constant capillary size across all model organisms (Assumption 7) by building the networks backwards starting with the capillaries and using the scaling relationships for vessel length (2) and radius (4) conforming with Assumptions 5 and 6, respectively. Given a group of organisms so constructed, we compute the group's scaling exponent with a linear regression of ln *N*_cap_ on ln *V*_blood_. An example of a regression for a particular group is shown in Figure 3A, justifying the assumptions underlying our approximation of the scaling exponent, Equation 17. To simulate a variety of taxa, we varied the size of the smallest organism in a group and chose the number of levels in the largest organism in that group to reflect a ratio *V*_blood,*L*_/*V*_blood,*S*_ as close as possible to the empirical value of 10^8^. (As a guide, the number of levels in an organism varies approximately logarithmically with body mass, such that the number of levels between the largest and smallest organism is approximately *N_L_*–*N_S_*≈3 ln (*M_L_*/*M_S_*)/4 ln(*n*) [1].) We used *n* = 2 as the most commonly observed branching ratio for the arterial system of mammals [51]–[54]. Many groups of model organisms were generated using this method, and in each case, a power-law provided an extremely good fit (*R* ^2^>0.99) to the data within a group, yielding a group-specific scaling exponent. We then plotted the dependency of these scaling exponents on the number of capillaries in the smallest organism of each group. This protocol accounts for effects that would be observed on the basis that the smallest organism in a group (taxon) sets the “small-organism-bias” contributed by this group to the overall statistic.

The results of these numerical calculations are shown in Figure 3B. Consistent with our analytical calculations, the scaling exponent drops below the asymptotic 3/4 limit with decreasing size of the smallest organism. As its smallest network decreases in size, a group contributes an increasing proportion of small networks, making the departure from the 3/4 exponent visible. It is clear from Figure 3B that Equation 17 is a good approximation for the deviations from the 3/4 law. We note that, despite a clear trend, the finite-size corrections to the scaling exponent are rather small and the approach to 3/4 is quite rapid. The corrections do not include 2/3 (the simple surface/volume scaling) even for networks with very few capillaries.

#### Networks with only area-increasing branching

As summarized in Assumption 6, area-increasing branching occurs not only as a consequence of minimizing dissipation in the regime of viscous flow but is also required for a portion of the network in order to match impedances for small vessels. Moreover, blood must slow down as it travels from the heart to the capillaries in order to allow for the efficient release and transfer of oxygen. By conservation of volume flow rate, the slowing of blood must be accomplished by area-increasing branching. Therefore, area-increasing branching has a significant influence even on the scaling exponent of groups dominated by large organisms. This influence only increases in groups biased towards smaller organisms in which a large fraction of the network exhibits area-increasing branching to minimize dissipation.

Here we analyze the limiting case of a network in which all levels are area increasing. This corresponds to a transition at *k̅* = −1, since the first area-increasing branching is level *k* = 0, so that *N̅* = *N*+1. The treatment of Equation 10 changes because *C*_0_ and *C*_1_ are no longer constant with respect to *N* (and thus *V*_blood_). However, returning to Equation 6 we note that only the second sum survives (*k̅* = 0), and this sum is just *N*+1, see Equation 8, rewarding us with a simplification of Equation 9
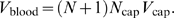
(18)The scaling relationship for this case is well approximated by a linear function. However, recall that *N*_cap_ = *n^N^*. Thus, *N* = ln *N*_cap_/ln *n* injects a logarithmic correction that slowly decays as larger mass (*V*_blood_) ranges are considered. As is evident from the expression for the tangent to the curve defined by Equation 18,
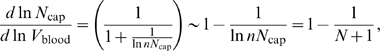
(19)the decay is slower than in the pure area-preserving case of the previous section, Equation 16, because of the logarithmic correction.

In complete analogy to section “Networks with only area-preserving branching”, we estimate the scaling exponent that would be measured for groups of organisms spanning a range of levels using only the difference in the logarithms of network volume and capillary number between the smallest and largest specimens. The leading-order expression is given by
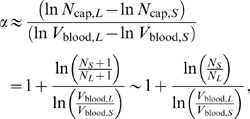
(20)where *N_S_* and *N_L_* are the number of levels in the smallest (*S*) and largest (*L*) organisms, respectively. Observe that ln(*N_S_*+1/*N_L_*+1) is negative because *N_S_*<*N_L_*. Hence, the scaling exponent *α* is less than 1 and approaches 1 from below in the limit of infinitely large organisms.

**Figure 4:**
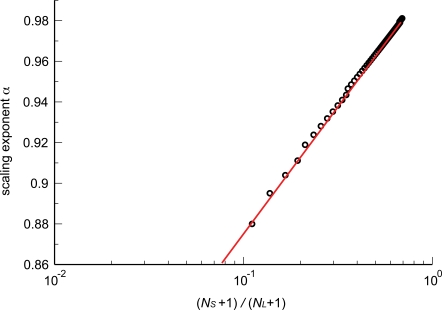
Finite-size corrections for networks with only area-increasing branching. As in Figure 3, but networks are now constructed with area-increasing branching only. The abscissa reflects the absolute size range, *N_S_*+1/*N_L_*+1, within each group used to determine the scaling exponent. *N_S_* and *N_L_* are the number of levels in the smallest and largest networks, respectively. Note that *N_S_*+1/*N_L_*+1 is always smaller than 1 and the scaling exponent *α* has an accumulation point at 1, the infinite size limit. Black circles: numerical data. Red line: analytical approximation, Equation 20.

As before, we constructed artificial datasets in accordance with WBE assumptions, but using the area-increasing relationship for vessel radii, Equation 3. The results of these numerical calculations are shown in Figure 4 for a branching ratio of *n* = 2. Consistent with the analytical calculation, the scaling exponent decreases as a greater proportion of small organisms are included within a group. The finite-size corrections are bigger than in the case of pure area-preserving branching, section “Networks with only area-preserving branching”. Likewise, the convergence to the infinite size limit, with an exponent of 1, is much slower in the area-increasing case than the convergence to the infinite size limit, with an exponent of 3/4, in the area-preserving case. The difference results from the approach scaling like 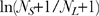 for area-preserving branching and like ln(*N_S_*+1/*N_L_*+1) for area-increasing branching. The abcissas in Figures 3 and 4 are chosen to reflect this difference. As can be seen in Figure 4, the analytical approximation, Equation 20, is remarkably accurate.

We conclude that finite-size effects on the scaling exponent are much more important for networks entirely composed of area-increasing branching than for networks operating entirely in the regime of area-preserving branching, described in section “Networks with only area-preserving branching”. The differential impact of finite-size effects in the two extreme cases is crucial for understanding finite-size effects in mixed networks with a transition between the two branching regimes.

#### Networks with a transition from area-preserving to area-increasing branching

The original WBE theory assumes that the cardiovascular system is a combination of area-preserving and area-increasing regimes. In large vessels, blood flow is predominantly pulsatile and the pulse wave can lose energy through reflections at vessel branch points. Minimizing this type of energy loss leads to the requirement that the total cross-sectional area of daughter vessels must preserve the area of the parent vessel in the early part of the network and switch to area-increasing branching farther downstream. In small vessels, on the other hand, blood flow is viscous. When optimizing energy expenditure for the viscous transport of blood, dissipation due to frictional drag from vessel walls becomes important. Minimizing such dissipation requires area-increasing branching, as summarized in Assumption 6, Equation 3. Optimizing these two flow regimes leads to a transition from area-preserving to area-increasing branching. WBE calculate the transition level to be *N̅* = 24 when *n* = 2 and *N̅* = 15 when *n* = 3. This transition level was determined as the level for which the impedance for reflections matches the impedance for dissipation. (A proper calculation of the transition, however, should equate the energy loss from reflections to that for dissipation while accounting for the attenuation of the wave.)

In the WBE model, these values for the transition level also set the size of the smallest organism, a mammal in which a heart beat cannot be sustained because the vessels are so small that the pulsatile flow is immediately dissipated. This is suggested in WBE by the assertion that “In a 3 g shrew, Poiseuille flow begins to dominate shortly beyond the aorta” [1]. West et al. (2002) [14] go further and actually calculate estimates for the size of the smallest mammal to about 1 g based on equating the transition level with the number of levels in the smallest organism. Consequently, the number of levels in the smallest mammal, a shrew, is taken to be *N_S_* = *N̅*+1 = 25 for a branching ratio of *n* = 2 and *N_S_* = *N̅*+1 = 16 for a branching ratio of *n* = 3. This allows the heart to be pulsatile, and the blood flow to become Poiseuille at the first level beyond the aorta.

The full form of Equation 10 describes this case. Recognizing that 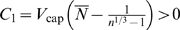 for our combinations of *N̅* and *n*, we can rewrite Equation 10 as
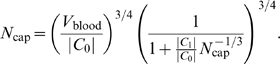
(21)


**Figure 5:**
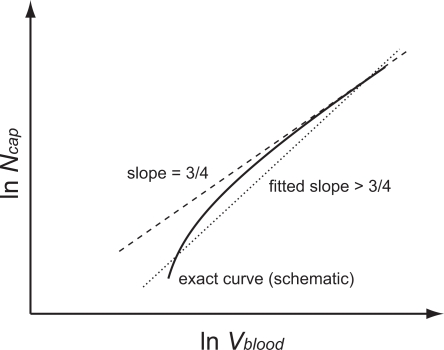
Schematic scaling relation for finite-size corrections in networks with both area-preserving and area-increasing branching. The dashed line schematically depicts the 3/4 power law of ln *N*_cap_ versus ln *V*_blood_ in the infinite network limit. The solid line dramatizes the curvature for the scaling relation that is obtained when the network has a transition point above which it has area-preserving branching and below which it has area-increasing branching. The dotted line illustrates the consequences of a linear regression on what is a curvilinear relationship that deviates below and away from the infinite-size limit, Equation 22. As a result, the WBE model predicts that fits to data for organisms whose vascular networks are built in mixed mode will yield scaling exponents that are larger than 3/4.

In comparison to Equation 15 the sign of *C*_1_ has changed, and this reverses, with significant consequences, our previous arguments. Specifically, as *N*_cap_ decreases, 
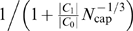
 now also decreases, resulting in smaller values of *N*_cap_ on the left of (21) than the values predicted from a pure 3/4 power-law. A log-log plot of this curve will asymptote to a straight line with a slope of 3/4 for large *N*_cap_ and will curve down and away from this asymptote as *N*_cap_ decreases. This effect derives from the fact that small mammals exhibit scaling exponents >3/4, a point raised by WBE in their original work [1]. Regressing a straight line on such a curvilinear relation will yield a scaling exponent above 3/4, as shown schematically in Figure 5. Indeed, the tangent to Equation 21 becomes steeper as *N*_cap_ decreases:
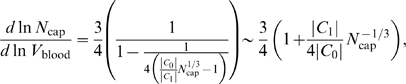
(22)where the last expression gives the leading-order correction for large *N*_cap_.

Using the same estimation procedure as in previous sections, we find that the scaling exponent computed over a given range of *N* is
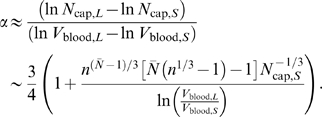
(23)Again, the dependence on *N_cap_*_,*S*_, which is tied to the number of levels in the smallest organism, captures absolute size effects. It makes the scaling exponent sensitive to the contributions from small organisms when groups span a range above their smallest member.

**Figure 6:**
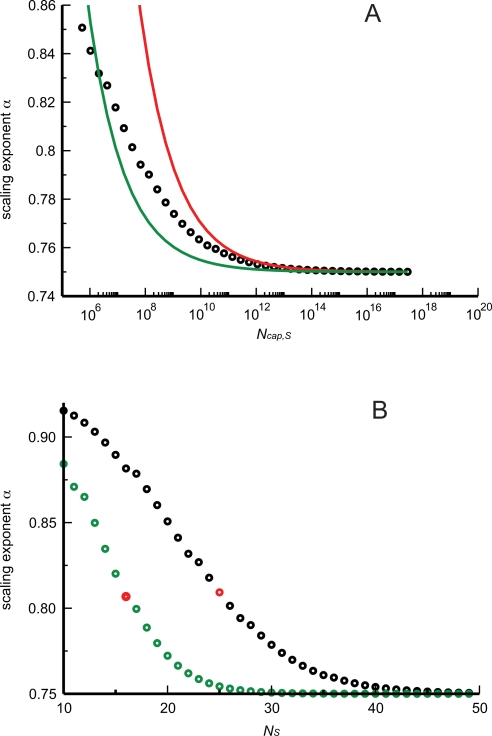
Finite-size corrections for networks with both area-preserving and area-increasing branching. (A) As in Figure 3B, we numerically determine the scaling exponent *α* by OLS regression within a group of artificial networks spanning roughly 8 orders of magnitude in body mass (blood volume). The exponent obtained from a group is plotted against the size of the smallest network in that group (as measured by the number of capillaries, *N*_cap,*S*_). Many groups are built by systematically increasing the size of the smallest network, resulting in the depicted graph. In all cases the branching ratio was *n* = 2. Black circles: numerical values. Red curve: analytical approximation, Equation 23. Green curve: Best fit to the shape of Equation 23, 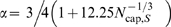. (B) As in (A), except that each exponent is plotted against the number of levels *N_S_* of the smallest network in the group from which it was determined. We display results obtained for a branching ratio *n* = 2 (black circles) and *n* = 3 (green circles). The red circles mark the predictions of the WBE model, since *N_S_* = 25 for the smallest network (a shrew, meaning *N̅* = 24 plus 1 level for pulsatile flow) in the case of *n* = 2, and *N_S_* = 16 for *n* = 3.

We generated artificial data as in the previous two sections, including a transition between area-preserving and area-increasing ratios of vessel radii, Equations 4 and 3, at *N̅* = 24 following WBE. The results of these numerical calculations for a branching ratio of *n* = 2 are presented in Figure 6A. We find that the fitted exponent decreases significantly as the size of the smallest organism included in the set increases. The figure also shows our analytical approximation, Equation 23, as well as a least-squares fit to the form of the equation.

In Figure 6B we replot the scaling exponent of a group against the number of levels of that group's smallest network (organism). Each group spanned eight orders of magnitude in body mass above its smallest member. We also included the curve obtained with branching ratio *n* = 3. We see that, once finite-size effects are accounted for, the WBE theory actually predicts a slope near 0.81 for *n* = 2 and *n* = 3 with the transition placed at *N̅* = 24 and *N̅* = 15, respectively.

As anticipated, the branching ratio has virtually no effect on the scaling exponent predicted by the WBE model. This is both because the leading-order term of 3/4 does not depend on *n* and because the first-order correction in Equation 23 depends on 
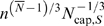
, which, for the WBE model, results in *n* ^(*N̅*−1)/3^ *n* ^−(*N̅*+1)/3^ = *n* ^−2/3^. Hence, the first-order correction becomes *n* ^−2/3^ *N̅*(*n* ^1/3^−1)/ln(*V*_blood,*L*_/*V*_bllod,*S*_), evidencing a very weak dependence on the branching ratio *n*. This explains the lack of a discernible difference in the scaling exponents for *n* = 2 and *n* = 3 in Figure 6B. However, different values for ln(*V*_blood,*L*_/*V*_blood,*S*_) or *N̅* will affect the predicted scaling exponent. Mammals are known to cover a size range, from a shrew to a whale, of about eight orders of magnitude. Within the WBE model, blood volume is directly proportional to body mass, which sets ln(*V*_blood,*L*_/*V*_blood,*S*_) = ln(10^8^)≈18.4 [1],[9]. The logarithm considerably weakens the dependence of the scaling exponent on blood volume, even for taxonomic groups that cover different size ranges. The calculated value for the transition level in the WBE model is 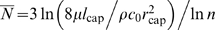, where *μ* denotes the blood viscosity, *ρ* the blood density, *c*_0_ the pulse wave velocity according to the Korteweg-Moens equation, and *r*_cap_ and *l*_cap_ are the mean radius and length of a capillary, respectively [1]. We study the effect of *N̅* on the scaling exponent in greater detail in section “Changing branching ratio across levels”.

The results of this section suggest that a strict test of the canonical WBE model should compare measured exponents to 0.81 rather than 3/4. Alternatively, one might argue that for the WBE model to yield a 3/4 exponent, the cardiovascular system of the smallest mammal must comprise many more than *N* = 25 levels, or the number of area-increasing levels must be much less than *N̅* = 25. It is unclear whether one of these options or some other modification of the model is biologically more realistic.

#### Summary of section “Finite-size corrections to 3/4 allometric scaling”

We find that finite-size effects change measured scaling exponents for networks with pure area preserving, pure area increasing or a mixture of both. Empirically determined scaling exponents for basal metabolic rate are typically a little lower than *α*≈3/4 [23]. Yet, the WBE model with finite-size corrections predicts an exponent of *α*≈0.81 (section “Networks with a transition from area-preserving to area-increasing branching”). Intriguingly, this is in closer agreement with empirical data for maximal metabolic rate [36] (but see also [37]). It is tempting to speculate whether the WBE model might not be more appropriate for organisms at their maximal rather than their basal metabolic state. However, if this were the case, we would expect the network to maximize power output rather than minimizing energy loss of transport, contradicting Assumption 6.

The exponent of *α*≈0.81 that we derived in section “Networks with a transition from area-preserving to area-increasing branching” for the finite-size corrected canonical WBE model is not within the 95% confidence intervals for any exponents determined from empirical data for metabolic rate in mammals [22],[23]. We are led to conclude that the WBE model is not fully supported by empirical data, assuming the data have been properly analyzed. One could hypothesize that adding more biological detail to WBE might yield a figure closer to the canonical value of 3/4. In the next section, we explore some of these issues.

### Making the WBE Model More Biologically Realistic

The discrepancies between the WBE model and data might be addressed in several ways: (i) by correcting for biases in the empirical distributions of species masses; (ii) by adding more detail to any of the WBE assumptions; (iii) by relaxing the assumptions. In Text S1 (Figure S4), we exemplify case (i) by accounting for the fact that most mammals, in particular those that have been measured, are of small mass. The body-size distribution across species is approximately log-normal. By sampling body sizes according to such a distribution and using the same numerical methods as in section “Finite-size corrections to 3/4 allometric scaling” above, we determined that the overall effect on the scaling exponent is essentially negligible (the exponent is slightly lowered). In section “Modifying the transition level between area-preserving and area-increasing regimes”, we illustrate approach (ii) by altering the level at which the transition from area-preserving to area-increasing branching occurs, as well as the width of the region over which it extends, as motivated by complexities in the hydrodynamics of blood flow. These considerations affect the scaling exponent, but the change is too small to restore the 3/4 figure. In section “Changing branching ratio across levels”, we illustrate approach (iii) by relaxing the assumption of a constant branching ratio (Assumption 4). We show that systematic changes in the branching ratio can significantly lower the measured scaling exponent and lead to intriguing non-linear effects that depend on where the transition from one branching ratio to another occurs.

#### Modifying the transition level between area-preserving and area-increasing regimes

The WBE approach determines the transition level from area-preserving to area-increasing branching by equating the impedance in regions with pulsatile flow with the impedance (resistance) in regions of smooth (Poisueille) flow [1]. Such a calculation results in an exact value for the vessel radius at which a match occurs. Because the capillary radius is assumed to be fixed across organisms (Assumption 7), a particular vessel radius translates into a particular level in the network hierarchy and a constant number of dissipation-minimizing levels in all organisms. WBE predict that this constant number of levels is *N̅* = 24 for a branching ratio of *n* = 2 [1]. WBE and West et al. [14] conclude that *N̅*+1 = 25 (one area-preserving level for impedance matching the pulse from the heart) is the number of levels in the smallest mammal, the shrew, and that humans have *N*≈34 levels, implying that most levels in humans exhibit area-increasing branching to minimize dissipation [1]. Indeed, most mammals are predicted to have significantly more levels with area-increasing than area-preserving branching. Although the contribution of the area-increasing regime is negligible in the limit of infinite mass, it has a significant effect on the predicted exponent for finite-sized mammals, as we showed in section “Networks with only area-increasing branching”, Figure 4.

There are reasons to doubt the assumptions behind WBE's calculation of the number of levels with area-increasing branching, *N̅*. First, it is unlikely that in the shrew pulsatile flow is immediately attenuated to smooth flow as soon as blood is pumped from the aorta. Second, the WBE calculation does not account for attenutation of the blood pulse as it travels across levels in the cardiovascular system. WBE assume a constant wave number and angular frequency, but these values likely change as blood flows across the system. Attenuation occurs because perfect impedance matching is difficult to achieve. In addition, attenuation certainly occurs in the transition region from impedance matching to dissipation minimization. Third, the transition should actually occur when the differential *energy loss* due to reflections is equivalent to the differential *energy loss* due to dissipation. This will yield a different prediction than merely equating the impedances, since the energy loss depends on constant pre-factors related to the blood viscosity, wave number, and other properties that differ in the two regimes. Fourth, the calculation of the transition level depends on radius and length of a capillary in a very sensitive manner. Reported values for both radius and length differ by as much as a factor of two, which introduces uncertainty into the WBE numbers. Fifth, the transition from impedance matching to dissipation minimizing is not a step function. Consequently, the transition between area-preserving and area-increasing branching is not a step function either. (Recall that impedance matching already requires a transition from area-preserving to area-increasing branching.) Although the transition may be rapid, it will likely take a few levels to occur.

**Figure 7:**
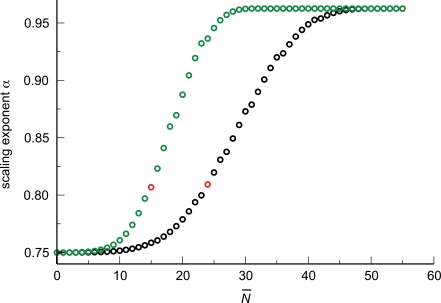
Influence of the location of the transition between area-preserving and area-increasing branching. The scaling exponent *α* is plotted against the number of levels *N̅* with area-increasing branching in the network. For each *N̅* the exponent was determined from a group of artificial networks that start from a smallest organism of fixed size and span eight orders of magnitude in blood volume to the largest organism, as described in section “Finite-size corrections to 3/4 allometric scaling”. *N̅* is varied from 0 (pure area-preserving branching) to the entire network (pure area-increasing). Black circles: Networks with branching ratio *n* = 2 and a smallest organism size of *N* = 25 levels. Green circles: Networks with branching ratio *n* = 3 and a smallest organism size of *N* = 16 levels. These graphs capture both finite-size effects and the effects of varying the extent of the network that is built with area-increasing branching. The exponent *α* changes from 3/4 to 1 as *N̅* grows, which is suggested by considering a composite of Figures 3B and 4. The red circles mark the prediction of the finite-size corrected WBE model (*N̅* = 24 for *n* = 2 and *N̅* = 15 for *n* = 3).

Addressing these problems will eventually require detailed hydrodynamical calculations and extensive knowledge of the cardiovascular system. We can, however, illustrate the effect of a change in *N̅* on the predicted scaling exponent. The finite-size corrections of section “Networks with a transition from area-preserving to area-increasing branching” crucially depend on the level at which the transition from area-preserving to area-increasing branching occurs. We used the same protocol of section “Finite-size corrections to 3/4 allometric scaling” to determine the dependency of the scaling exponent on *N̅*. To do this, we fix the size of the smallest organism under consideration; in this case we choose to set *N_S_* = 25 levels for consistency with the results from the canonical WBE model. We then construct a group of model organisms for each value of *N̅*. When *N̅* = 0 we have the case of pure area-preserving branching (section “Networks with only area-preserving branching”) and when *N̅* = 24 we have the result of the canonical WBE model for *n* = 2. When *N̅*>25 some of the organisms in the set will have networks consisting entirely of area-increasing branching (corresponding to networks of the type considered in section “Networks with only area-increasing branching”). In every case the size of the largest organism was chosen such that it exhibited a *V*_blood_ that is 8 orders of magnitude larger than that of the smallest organism. These results are summarized in Figure 7. Consistent with the analytical calculation, the scaling exponent increases as more organisms with area-increasing branching are included in the analysis. Again, the scaling exponents obtained within the canonical WBE model are significantly larger than the 3/4 figure (and sometimes lower figures) determined from empirical data.

**Figure 8:**
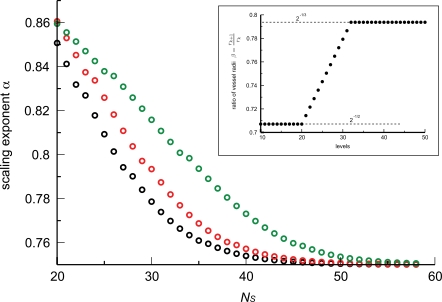
Influence of an extended transition region. The three curves are analogous to those in Figure 6B. (In fact, the black curves are identical.) The figure shows how a transition over 12 (red circles) and 24 levels (green circles) shifts the curve relative to the WBE assumption of a transition over a single level (black circles). The more extended the transition region, the fewer the levels built with area-preserving branching. The scaling exponent increases as a consequence. Inset: The linear interpolation of *r_k_*_+1_/*r_k_* as a function of network level used in generating the red curve. The transition occurs from *β* = 2^−1/2^ = 0.707 to *β* = 2^−1/3^ = 0.794 over 12 levels centered at the WBE transition level *N̅* = 24.

We also examined the consequences of a transition region, rather than a single transition level, from area-preserving to area-increasing branching. We spread the transition out over *k*′ levels centered at the transition in the WBE model. The ratio of the radii at levels within the transition region is determined by linear extrapolation between Equations 4 and 3, see inset of Figure 8. The scaling exponent as a function of network size is shown in Figure 8 for various transition depths *k*′. Remarkably, this more gradual transition has little effect on the predicted exponent.

#### Changing branching ratio across levels

All calculations thus far assume that the branching ratio *n* is constant. It is quite likely, however, that a vessel does not always branch into the same number of daughter vessels regardless of whether the branching occurs at levels close to the heart or close to the capillaries. For example, according to Zamir [51],[54], cardiovascular systems have a branching ratio of *n* = 2 near the aorta, but earlier research by Horsfield [67] suggests a branching ratio closer to *n* = 3 or even slightly greater for smaller vessels, including those near the capillaries. To investigate the consequences of a changing branching ratio, we construct networks for which *n* = 2 up to some level at which a transition occurs to a greater value, such as *n* = 3, 4, 5. We define *N̅* *_b_* in analogy to *N̅* for the transition from area-preserving branching to area-increasing branching discussed above. In this case, *N̅* *_b_* defines the constant number of levels between the capillaries and the branching ratio transition. That is, if *N̅* *_b_* is equal to 10, then all the networks employ 3-, 4- or 5-branching for the 10 levels closest to and including the capillaries and 2-branching from that point to level 0 (the heart). In these computations we include a transition from area-preserving to area-increasing that we assume to occur in one step (as in the WBE model). Given that the branching ratio changes somewhere in the network, however, we can no longer assume that this transition will occur at a constant number of levels from the capillaries. That is, it will occur 24 levels from the capillaries in the case of pure 2-branching, but it will occur 15 levels from the capillaries if all of those levels are 3-branching and it will occur at some other level for a mixture. To overcome the inherent dependence of the radial scaling transition on *N̅* *_b_*, we calculated the transition level using the WBE formula [1]: 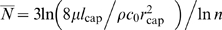, where *μ* denotes the blood viscosity, *ρ* the blood density, and *c*_0_ the pulse wave velocity according to the Korteweg-Moens equation (for definitions see, for example, [68]). The vessel radius at the transition is then given by 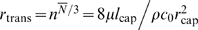. We obtain *r*_trans_≈1 mm, using the same numbers as WBE, that is, *μ* = 0.004 Ns/m^2^, *l*_cap_ = 0.08 mm, *ρ* = 1050 kg/m^3^, *c*_0_ = 600 cm/s, *r*_cap_ = 4 µm. As argued above, there are reasons to doubt this calculation for the radial scaling transition. We employ the same formula, however, since that allows us to make a direct comparison with the canonical WBE model and lets us consider just the effects of changing the branching ratio.

As before, networks for the changing-*n* model were built backwards from the capillaries using area-increasing branching. After *N̅* *_b_* levels, the branching ratio was changed from its downstream value (*n* = 3, 4, 5) to 2. Whenever the radius of a vessel exceeded the cutoff radius for *r*_trans_, branching changed from area-increasing to area-preserving. We held constant the number of levels, *N*, in the smallest organism for all the values of *N̅* *_b_*. For these calculations we set this number to be 25 and varied the size of the largest organism so that each point represented a dataset that spans 8 orders of magnitude in *V*_blood_.

**Figure 9:**
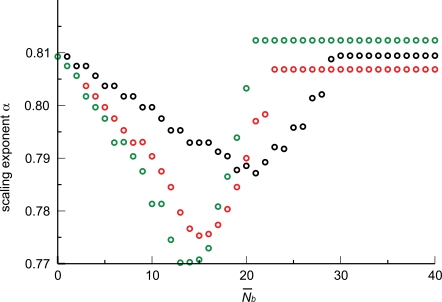
Influence of the branching ratio. The scaling exponent *α* as a function of the number of levels *N̅* *_b_* at which the branching ratio switches from *n* = 2 to the indicated value of 3 (black circles), 4 (red circles) or 5 (green circles). *N̅* *_b_* varied from 0 (a branching ratio of *n* = 2 at all levels) to the depth of the entire network (a branching ratio of *n* = 3, 4, or 5 at all levels). As in Figure 8, each exponent was calculated from networks that spanned eight orders of magnitude in blood volume. In these calculations, network levels with vessel radii ≤1 mm were built according to area-increasing branching, while vessels with radii larger than 1 mm followed area-preserving branching. These curves correspond to a cardiovascular system in which the branching ratio *n* is smaller near the heart and larger toward the capillaries. In all cases, a change in branching ratio within the network decreases the predicted scaling exponent, bringing it closer to the empirical value of 3/4 without ever touching it.

As shown in Figure 9, a change in the branching ratio within the same network yields exponents that are always smaller than for networks with a constant branching ratio. The exact value depends non-monotonically on the level at which the branching transition occurs. We conclude that finite-size effects for networks with varying branching ratio lowers the exponents in the direction toward the empirical value of about 3/4. Still, our numerical calculations never equal or drop below 3/4. Measurements of actual branching ratios should become increasingly feasible using plasticene casts and advanced imaging techniques. Such data would enable a better parameterization of this particular extension of the WBE model.

### Comparison to Empirical Data

**Figure 10:**
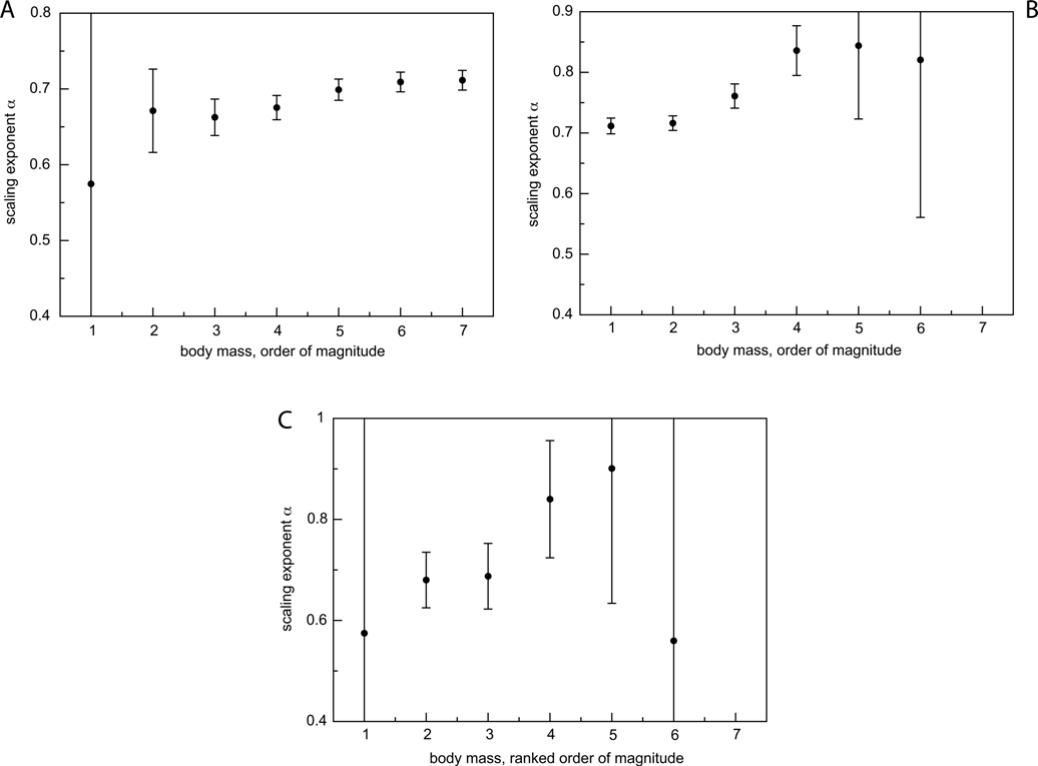
Dependency of the scaling exponent on body mass range as determined by ordinary least squares regression on empirical data. The data are binned in orders of magnitude for body mass as described in the text. (A) Cumulative binning starting with smallest mammals. (B) Cumulative binning starting from largest mammals. (C) Exponents from individual order-of-magnitude bins. The exponents computed from these aggregations of empirical data vary both above and below 3/4. Note, however, that in all cases the allometric exponents tend to increase with increasing body mass. The error bars represent the 95% confidence intervals. When data is scarce, the confidence intervals become so large that the exponents cannot be trusted. (The full range of some error bars is cut off by the scale of the plots.)

Savage et al. [23] published an extensive compilation of empirical data for basal metabolic rate and body mass of 626 mammals. In this section we compare the dependency of scaling exponents on body mass as obtained from this dataset to our predictions for scaling exponents with finite-size corrections. We sorted organisms according to body mass and grouped them, starting with the smallest exemplar, into disjoint bins spanning one order of magnitude each. We then analyzed this data compilation in three ways. First, we determined the scaling exponents for successive cumulations of bins. At each addition of a bin, we computed a linear regression on the entire cumulated data, plotting the resultant scaling exponent against the range of sizes. In other words, the first scaling exponent is determined for the first order of magnitude in body mass, the second exponent is determined for the first two orders of magnitude, and so on. This is similar in spirit to the procedure used for analyzing and presenting the numerical data in section “Finite-size corrections to 3/4 allometric scaling”. The result is shown in Figure 10A. In a second approach we proceeded similarly, but starting with the largest order of magnitude in body mass, then successively adding bins of smaller orders (Figure 10B). Lastly, we computed the scaling exponent for each bin separately (Figure 10C).

The panels of Figure 10 show the results with error bars based on the 95% confidence intervals obtained from ordinary least squares (OLS). In panels 10A and 10B, the exponents exhibit an increasing trend with body mass. Panel 10C shows a similar trend for bins that correspond to intermediate mass ranges. These are the bins that contain most of the data points. There is too much scatter at either end of the body mass distribution to make a statement about the entire range for panel 10C. We find that for those ranges and aggregations with smallest scatter (as determined from error bars), the scaling exponent approaches the 3/4 figure from below. Although these data are suggestive, it would be incautious at this point to assert that the data flatten out at 3/4 for some maximum mammalian size. Given the current dataset, however, an “asymptotic” 3/4 scaling seems a reasonable guide.

The concave increase of the scaling exponent with body mass is most consistent with a finite-size WBE model based on pure area-preserving branching throughout the network, see section “Networks with only area-preserving branching”. (The concave increase of the scaling exponent, Figure 3B, corresponds to a convex relationship between metabolic rate and body mass, see the schematic in Figure 2.) Recall that in our numerical studies of section “Networks with only area-preserving branching” the scaling exponent approached 3/4 in a concave fashion from below, while networks built entirely with area-increasing branching (section “Networks with only area-increasing branching”) have scaling exponents that always lie above 3/4, converging to an accumulation point at 1. Networks built with a mixture of these branchings (section “Networks with a transition from area-preserving to area-increasing branching”), approach 3/4 scaling in a convex fashion from above, opposite to the trends seen in Figure 10. (The convex decrease of the scaling exponent, Figure 6A, corresponds to a concave relationship between metabolic rate and body mass, see the schematic in Figure 5.)

A similar analysis of a more limited dataset for heart rate (26 data points) and respiratory rate (22 data points) [23] also shows a trend that is not easily reconciled with our finite-size corrections for networks with a mixture of area-preserving and area-increasing branching. In WBE, heart rate *ω* and respiratory rate *R* are both predicted to scale as *ω*∝*R*∝*M* ^−*α*/3^ (see Table S1 and related text in section “Impact of finite-size corrections on additional WBE predictions” of Text S1). Since our calculations in section “Networks with a transition from area-preserving to area-increasing branching” yield scaling exponents, *α*, that approach 3/4 from above as body mass increases, we expect the scaling of heart and respiratory rates to both have exponents that are bounded by the maximum value of −1/4. The WBE model with finite-size corrections predicts *α*≈0.81. Hence, heart and respiratory rates should scale as *M* ^−0.27^ and asymptote to −1/4 with increasing mass. That is, there should be very little change in the scaling exponent when analyzing data for either small or large mammals. This does not match empirical heart rate data well. Regressing on the first three, four, and six orders of magnitude in body mass yields exponents of −0.33, −0.27, and −0.25, respectively. The match is worse for respiratory rate data. Regressing on the first two, three, five, and seven orders of magnitude in body mass gives exponents of −0.64, −0.44, −0.34, and −0.26, respectively. We observe a convergence to −1/4, but over a much larger range of scaling exponents than expected.

While the WBE model has been predominantly interpreted in the context of interspecific scaling [9],[23], metabolic rate also varies with body mass during development. Such *intra*specific data [29],[69] sometimes exhibits a concave curvature across growth stages ranging from young to adult mammals. Indeed, our finite-size corrections for the canonical WBE model predict a concave curvature of ln *B* versus ln *M*. However, they also predict an asymptotic approach to a slope of 3/4 for large mammals, and thus a fitted OLS slope for the entire body mass range that is greater than 3/4, as schematically shown in Figure 5. In his Table 5, Glazier [29] reports slopes from 29 intraspecific regressions for 14 species of mammals. From these, we compute an average slope *α* = 0.70; in this dataset, 20 of the slopes are smaller than 3/4 and only 9 of the slopes are larger than 3/4. This is inconsistent with our predictions. Moreover, the average body mass range of mammals, for which Glazier reports intraspecific regressions, spans only half an order of magnitude. Yet, our calculations show that several orders of magnitude in body mass are required to detect curvilinearity from finite-size effects, as seen in Figure 6A. We thus conclude that the curvature revealed by these intraspecific datasets is either unrelated to finite-size effects or fails to support the finite-size corrected canonical WBE model.

It is important to note that empirical data for the inter- and intraspecific case (especially for restricted size classes) are rather limited. We therefore do not wish to overstate the strength of our conclusions. We merely report discrepancies between the predictions of the canonical WBE model and limited sets of data. We anticipate that further data acquisition, statistical analysis, and model refinement will bring theory and data into agreement.

## Discussion

Over the past decade, the WBE model has initiated a paradigm shift in allometric scaling that has led to new applications (e.g., [2],[70],[71]), new measurements and the refinement of data analysis (e.g., [41]–[53]), and the recognition of connections between several variables that describe organismic physiology [1],[23]. However, WBE has also drawn intense criticism and sparked a heated debate [15]–[28].

In section “Assumptions of the WBE model”, we provide a detailed presentation of the complete set of assumptions and calculations defining the WBE model. While none of these originated with us, the literature lacked, surprisingly, an exhaustive exposition. (In particular, the consequences of Assumption 6 are a distillation of hydrodynamical calculations that we summarize in Text S1.) In section “Derivation of the 3/4 scaling exponent”, we connect each step in the derivation of the main WBE result to the assumptions it invokes. In this way, we provide a self-contained platform for motivating, deriving, and interpreting our results.

One of our main objectives is to clarify that the WBE model predicts (and thus “explains”) the 3/4 exponent of the scaling law relating whole-organism metabolic rate to body mass *only* as the limit of infinite network size, body mass, and metabolic rate is approached. Although this fact was appreciated by WBE in their original work [1] the nature of this approximation has been broadly misunderstood in the subsequent literature, e.g., [16],[29]. In this work, we conduct a systematic exploration of finite-size effects in the WBE framework and find that these effects yield measurable deviations from the canonical 3/4 scaling exponent, shifting the actual prediction to a value closer to 0.81 when published parameters are employed [1],[14]. This finding has major implications and immediately clarifies some contentious issues. On the one hand, the common criticism that the WBE model can only predict a scaling exponent of 3/4 is incorrect. As we show in section “Finite-size corrections to 3/4 allometric scaling”, a continuum of exponents can be obtained as a function of body-mass. On the other hand, the 0.81 figure (obtained for *N̅* = 24 and *n* = 2) shifts the predicted exponent for mammals away from the canonical figure of 3/4 that reflects current data analysis. In section “Impact of finite-size corrections on additional WBE predictions” of Text S1 we report the finite-size corrections for several variables related to vascular physiology that were documented in the original WBE paper [1].

A major consequence of the curvilinear relationship between ln *B* and ln *M* predicted by the model is the fact that the scaling exponent, as measured by a simple power law regression, will show a dependence on the absolute masses of the organisms in question. Notably, our numerical calculations for area-increasing branching in Figure 4 are consistent with the linear scaling of metabolic rate versus body mass that has been observed for small fish [32]. Indeed, with minor modifications, our Equations 19 and 20 could be used to test the form of isometric scaling observed in young and small fish. It should be noted, however, that the magnitude of these finite-size corrections depends strongly on certain network properties, such as *N̅*.

Furthermore, we find evidence for size-dependent relationships in the available empirical data for mammals (section “Comparison to empirical data”). Specifically, we find that the measured scaling exponent tends to *increase* with body mass, indicating that the empirical data (of log metabolic rate versus log body mass, or, equivalently, ln *N*_cap_ versus ln *V*_blood_) exhibits convex curvature (i.e., the type of relationship dramatized in Figure 2). However, networks constructed with a *mixture* of area-increasing and area-preserving branching can *never* produce scaling relationships with exponents less than 3/4 and, although 3/4 scaling is approached in the limit of networks of infinite size, the exponents always approach 3/4 from above (unlike in Figure 10). Mixed networks of this type display inherently concave curvature of the log metabolic rate versus log body mass relationship (i.e., the type of relationship dramatized in Figure 5). That is, a group of organisms of larger sizes will yield smaller fitted exponents than a group of organisms of smaller sizes. Yet, empirical data are best fit by a power law with an exponent less than 3/4 and demonstrate convex curvature in several datasets of log metabolic rate versus log body mass. *Thus*, *assuming that this represents the actual curvature in nature*, either (i) a transition between radial scaling regimes does not occur, potentially contradicting Assumption 6 of the WBE model, or (ii) at least one assumption of the WBE model must be modified.

The case for pure area-increasing branching (hypothesis (i) above) within the WBE model is somewhat problematic. The only way for such a network to be consistent with Assumption 6 would be to posit that the transition from area-preserving to area-increasing regimes occurs at a vessel radius *smaller* than a capillary; in this case, this transition would in principle exist but would simply never actually be observed in nature. A number of facts contradict this explanation. For one, estimates place the transition at vessel radii of about 1 mm. Despite the fact that predictions of where the transition might occur are problematic (see section “Modifying the transition level between area-preserving and area-increasing regimes”), the estimate is unlikely to be 3 orders of magnitude larger than the actual value (since capillary radii are on the order of 1 µm in radius). A further complication is that a pure area-preserving network would theoretically not be able to “slow down” blood flow due to the conservation of volume flow rate for an incompressible fluid. The fact that blood flows much more quickly in the aorta than it does in the capillaries would tend to argue that area-increasing branching must occur *somewhere* in the network. Finally, there is the simple fact of Murray's Law; empirical findings squarely place *β* for small vessels in the neighborhood of *n* ^−1/3^, strongly implying that area-increasing branching is in fact dominant when vessel radii are small [54],[57].

In our hands, empirical data seem most consistent with networks built with purely area-preserving branching, although the lack of very high-quality data for both metabolic rate and body mass makes it difficult to be absolutely certain of this trend. The reasoning outlined above makes hypothesis (i) appear somewhat unlikely. This leaves us with a riddle: cardiovascular networks with architectures that support the scaling trends observed for real organisms would seem to violate Assumption 6 of the WBE model. We are thus led to believe that some modification of assumptions 2–8 is necessary to explain the concavity in the data and an empirical scaling exponent less than 3/4. While a model that aligns with the empirical evidence might differ from the canonical WBE model (assumptions 2–8 plus specific values for the parameters *N̅* and *n*), we believe such a model will squarely remain within the WBE framework (assumption 1, that is, the exploration of allometric scaling in the context of resource distribution networks).

Resolving this paradox will likely require intensive further data analysis and extension of the canonical WBE model. It is clear that work in this area would benefit from a more detailed empirical understanding of cardiovascular networks themselves. Although data for the coronary artery in humans, rats, and pigs exist [51]–[54],[72],[73], along with measurements for the vascular system in the lungs of armadillos [74], stringent tests of the core WBE assumptions require measurements throughout the body, in a larger variety of species, and for vessels farther away from the heart. Measurements are needed especially for the number of levels from the heart to the capillaries for different species, the scaling ratios of vessel radii (*β* = *r_k_*_+1_/*r_k_*) and vessel lengths (*γ* = *l_k_*_+1_/*l_k_*), vessel blood flow rates, and branching ratios (*n* = *N_k_*_+1_/*N_k_*). Such data will help to assess the extent to which mammalian vascular systems are space filling (Assumption 5), the scope of area-preserving and area-increasing branching (Assumption 6), the value(s) of the branching ratio throughout the network (Assumption 4), and the degree of symmetry or asymmetry in branchings and scaling ratios (Assumption 3). Analyzing intraspecific variation in network geometry may also enable a quantification of selection pressures for optimality with respect to energy loss, as implied by Assumption 6 (see Figures S1 and S2 in Text S1). Advances in fluorescent microspheres [74], plasticene casting, imaging, and image analysis all hold promise for a careful gauging of the vascular system.

In this paper we have begun the process of relaxing some assumptions of the canonical model. Although these modifications produce interesting results, they do not fully address the riddles discussed above. Addition of further biological realism, such as asymmetric branching or the flow characteristics of the slurry of blood cells at small vessel sizes, may generalize the WBE model from an asymptotic predictor of metabolic scaling into a universal theory that provides an understanding of which properties of resource distribution networks are most relevant for metabolic scaling in any given biological context. This will enable testing the very soundness of the WBE framework (Assumption 1) and the extent to which the cardiovascular system shapes one of the most wide ranging regularities across animal diversity.

## Supporting Information

Figure S1 Minimizing energy loss to dissipation. Plot for an arbitrary level of the cardiovascular network (*l/*(*n^k^r* ^4^)+*λ*∼*n^k^r* ^2^ *l*) taken from the first two terms of the objective function *P* in Equation 4 versus the the radius of the vessel, *r*. For simplicity, we chose the parameters *l* = 1, *r* = 1, and *k* = 5. Using these parameters, the predicted value for the Lagrange multiplier is *λ*∼ = 2/*n* ^2*k*^ *r* ^6^ = 1/512≈0.00195. We plot the first two terms of the objective function versus *r* for the choices of the Lagrange multiplier *λ*∼ at 1/512≈0.00195 (red curve) and show that the minimum does indeed occur at the chosen value of *r* = 1. We also show results for the same choice of parameters as before but with *λ*∼ chosen to be 0.0015 (black curve) and 0.0025 (green curve) respectively. These other values for *λ*∼ do not exhibit a minimum at *r* = 1,indicating that either they are not evolutionarily optimized or that the objective function constructed by WBE is incorrect.
(0.46 MB EPS)

Figure S2 Minimizing energy loss to reflection. Plot of the reflection coefficient squared (|*R*|^2^ = |(1−*nZ_k_*/*Z_k_*_+1_)/(1+*nZ_k_*/*Z_k_*_+1_)|^2^) versus the ratio of vessel radii *β* = *r_k_*_+1_/*r_k_* at a branching junction. The impedances are defined as in Equations 34 and 35 in the supplementary material. The kinematic viscosity, the ratio of blood viscosity to blood density, is 2.57×10^−6^ s/m^2^. We choose a bifurcating branching ratio *n* = 2, and the wave frequency and the radius of the parent vessel are taken to be 1 Hz and 1.5 cm, respectively, to approximate the values for the human aorta. As long as the radius of the parent vessel is large as defined by Equation 33, different choices for the wave frequency and radius of the parent vessel will change the exact values in the plot but not the shape of the curve. The plot reveals that the reflection coefficient is zero at *β* = 0.707≈2^−1^/^2^, which exactly corresponds to area-preserving branching and impedance matching.
(0.41 MB EPS)

Figure S3 Minimizing total power loss. The graph depicts the *β_k_*≡*r_k_*_+1_/*r_k_* that minimizes the total power lost in going from one branching level to the next as a function of the vessel radius, *r_k_*, in units of meters. We have chosen *n* = 2, the minimum radius to be the average value for a capillary, *r_cap_* = 8 µm, and the physical values for kinematic viscosity of *μ*/*ρ* = 2.57×10^−6^ s/m^2^ and for wave frequency of *ω*_0_ = 1.17 s^−1^. For smaller values of *r_k_*, the minimum occurs around *β_k_* = 0.794, and for larger values of *r_k_*, the minimum occurs around *β_k_* = 0.710. These values match the predicted values from WBE of *β_k_* = *n* ^−1/3^ = 2^−1/3^ = 0.794 and *β_k_* = *n* ^−1/2^ = 2^−1/2^ = 0.707 extremely well. Also, note that the transition from area-preserving to area-increasing branching begins at *r_k_*≈1 cm and is completed by *r_k_*≈1 mm.
(0.41 MB EPS)

Figure S4 Log-normal sampling bias for small networks. A single realization of 1000 numerically generated data points for networks with a branching ratio of *n* = 2 built with area-preserving branching for large vessels and area-increasing branching for small vessels. The transition between these regimes is always *N̅* = 24. The networks are generated by sampling the number of levels from a log-normal distribution. (See text for details.) Based on empirical data [16], the underlying distribution ranges from a minimum of 27 levels to a maximum of 44 levels with an average of 32 and a standard deviation of 4. The scatter is generated by multiplying the blood volume and number of capillaries by two random numbers drawn from a uniform distribution on the interval [0.3,1.7]. This procedure generates one order of magnitude scatter in metabolic rate for a given mass, mimicking the variation observed in empirical data for mammals. Red line: Fit to artificial data without scatter-exponent is 0.83. Green line: Fit to artificial data with added scatter-exponent is 0.8.
(0.78 MB EPS)

Text S1 Sizing Up Allometric Scaling Theory
(0.39 MB PDF)
