# Supplementary material for: Sizing Up Allometric Scaling Theory
Source: PLoS Comput Biol. 2008 Sep 12;4(9):e1000171. doi: 10.1371/journal.pcbi.1000171 (PMC2518954; doi:10.1371/journal.pcbi.1000171)
Supplement: Text S1 — Sizing Up Allometric Scaling Theory (0.39 MB PDF) [file pcbi.1000171.s005.pdf]

Text S1

Van M. Savage, Eric J. Deeds and Walter Fontana

Department of Systems Biology, Harvard Medical School, 200 Longwood Avenue, Boston MA 02115, USA

Email: Van Savage - van_savage@hms.harvard.edu; Eric Deeds - deeds@hms.harvard.edu;
Walter Fontana- walter@hms.harvard.edu;

Energy loss in vascular networks

In the following three sections, we summarize the hydrodynamic reasoning that provides the formal background to various Assumptions gathered in section 2.1 of the main text, in particular the area-preserving and area-increasing constraints, equations (3) and (4), as well as the proportionality of blood volume and body mass. As we repeatedly emphasize, this material is not novel. Our intent is to provide a concise and rigorous summary of the WBE theory that is scattered across the literature.

Minimization of energy loss to dissipation

Lagrange multiplier calculation

Assumption 6 of West, Brown, and Enquist (WBE) posits that natural selection has minimized the energy required for pumping blood through the vascular network. The benefit, presumably, is to free up as much energy as possible for other actions that might affect fitness, such as foraging, growth, and reproduction [1, 2]. In this section, we focus on minimizing power lost to dissipation that results from friction between blood and vessel walls. Friction becomes important when a large proportion of the blood is in contact with vessels walls. This dominant form of energy loss in small vessels. Specifically, we wish to minimize the power lost to dissipation by flowing blood through a network that is

(i) of fixed size

(ii) contained within the body volume, and

(iii) space-filling, as defined in Assumption 5 of the main text.

Without these requirements, the calculation would merely end up revealing that power is minimized either by not pumping the blood at all or by letting vessel radii become infinite - in either case the friction is zero.

In the subsequent calculation, power loss does not vary with time. Minimizing power loss is, therefore, the same as minimizing energy loss - the work needed to pump blood for some fixed amount of time. Owing to requirement (i), the calculation minimizes energy loss for any organism of fixed size. Although this will constrain the architecture of a network, it does not yet connect organisms of different body masses. For this we need to establish a connection between network size or volume (i.e., blood volume) and body mass, which we shall do in section "Proportionality of blood volume and body mass".

The power lost in the cardiovascular system to dissipation is given by $P = \dot{Q}_0^2 Z_{net}$, where $\dot{Q}_0$ is the volume flow rate of blood and Z_{net} is the total impedance (resistance) to blood flow in the network. To minimize the power loss subject to constraints (i)-(iii), we use the method of Lagrange multipliers [3]. The objective function is given by

$$\mathcal{P} = \dot{Q}_0^2 Z_{net} + \lambda V_{blood} + \lambda_M M + \sum_{k=0}^{N} \lambda_k n^k l_k^3, \tag{1}$$

where the last three terms represent the requirements (i), (ii), and (iii), in this order [1,2]. Each constraint appears with a corresponding Lagrange multiplier: λ, λ_M, and $\lambda_k, k = 1, \ldots, N$. The space filling requirement leads to N constraints, because each level k must separately be space filling (see Assumption 5 in the main text). The terms in the last sum of equation (1) make use of Assumption 4 that the branching ratio is constant, and thus the number of vessels at level k is n^k.

Our goal is to understand how minimization of energy dissipation constrains the network architecture. To this end, we express the objective function (1) explicitly in terms of network parameters. In particular, we know from the main text that $V_{blood} = \sum_{k=0}^{N} N_k \pi r_k^2 l_k$. The impedance for smooth flow through a rigid vessel at level k is given by the Poiseuille formula for resistance

$$Z_k = 8\mu l_k / \pi r_k^4, \tag{2}$$

where μ is the viscosity of blood [4–7]. Within each level, the vessels are in parallel, thereby lowering the impedance to flow through a single vessel. The total impedance at level k is the impedance for a single vessel divided by the total number of vessels, $Z_{k,tot} = 8\mu l_k / n^k \pi r_k^4$. Across levels, the vessels are in series, thereby compounding the resistances of individual vessels, making it harder to push blood to the next level. Thus, the total resistance across the whole network is the sum over the impedances at each level, $Z_{net} = \sum_{k=0}^{N} 8\mu l_k / n^k \pi r_k^4$ [1,2]. Combining these expressions and absorbing all constants into new definitions of the Lagrange multipliers (e.g., $\lambda' = \pi\lambda$) yields

$$\mathcal{P} = \dot{Q}_0^2 \sum_{k=0}^{N} \frac{8\mu l_k}{n^k \pi r_k^4} + \lambda' \sum_{k=0}^{N} n^k r_k^2 l_k + \lambda'_M M + \sum_{k=0}^{N} \lambda'_k n^k l_k^3. \tag{3}$$

Since the Lagrange multiplier calculation consists in differentiating this function and setting the result to zero, we can divide the equation by any constant without affecting the outcome. We divide by $8\mu\dot{Q}_0^2/\pi$ (since $\dot{Q}_0^2$ is constant by assumption), and absorb residual constants into a new set of Lagrange multipliers, which we denote with $\tilde{\lambda}_\bullet$, where the bullet is a placeholder for an

appropriate subscript:

$$\mathcal{P} = \sum_{k=0}^{N} \frac{l_k}{n^k r_k^4} + \tilde{\lambda} \sum_{k=0}^{N} n^k r_k^2 l_k + \tilde{\lambda}_M M + \sum_{k=0}^{N} \tilde{\lambda}_k n^k l_k^3. \tag{4}$$

To determine what the minimization of $\mathcal{P}$ implies for the vessel radii, we proceed with the usual Lagrange-multiplier calculation by differentiating $\mathcal{P}$ with respect to r_k, at an arbitrary level k, and setting the result to zero:

$$\frac{\partial \mathcal{P}}{\partial r_k} = -\frac{4 l_k}{n^k r_k^5} + 2\tilde{\lambda} n^k r_k l_k = 0. \tag{5}$$

Solving this equation for the Lagrange-multiplier $\tilde{\lambda}$ yields

$$\tilde{\lambda} = \frac{2}{n^{2k} r_k^6}. \tag{6}$$

For the blood volume to be space filling (Assumption 5) and to conform with body mass, M, $\tilde{\lambda}$ must be independent of k, which requires that $n^{2k} r_k^6$ be constant across levels k. This, in turn, implies that $n^{2(k+1)} r_{k+1}^6 / n^{2k} r_k^6 = 1$ or the WBE result [1,2] that

$$\beta_> = \frac{r_{k+1}}{r_k} = n^{-1/3}, \tag{7}$$

which is equation (3) in Assumption 6 of the main paper. Such a scaling ratio for radii means that the total cross sectional area of all vessels at level k, $A_{k,tot}$ is less than the total cross sectional area at level $k+1$, $A_{k+1,tot}$. Indeed, using equation (7), we obtain $A_{k,tot} = n^k \pi r_k^2 = n^{k+2/3} \pi r_{k+1}^2 = n^{-1/3} A_{k+1,tot}$. This area-increasing property causes the blood to slow down as it flows through the network, which not only reduces resistance from friction, but also allows for effective transfer of oxygen from capillaries to cells by diffusion.

To examine the strength of selection on these optimal choices for $\tilde{\lambda}$ and the radii scaling ratio, $\beta = r_{k+1}/r_k$, one needs to understand how variation in these parameters affects the energy minimization relative to constraints and thus the objective function $\mathcal{P}$. To describe how this could be done, we take just the terms of $\mathcal{P}$ that depend on radius at a given level k, $l_k/(n^k r_k^4) + \tilde{\lambda} n^k r_k^2 l_k$. These terms represent the energy loss to dissipation for a specified volume of blood that must be pumped through level k. Separately minimizing each level k guarantees that the sum over levels will be minimized because it is the sum of all positive terms that are at their minimum. The two other terms in $\mathcal{P}$, $\tilde{\lambda}_M M + \tilde{\lambda}_k n^k l_k^3$, can be ignored because they will just add a constant value that is independent of r_k and that does not affect the curvature of $\mathcal{P}$ for varying r_k. Furthermore, for this calculation the choice of multiplicative constants is left arbitrary and should not affect the curvature. It is difficult to display the results or to provide a visual intuition for Lagrange multiplier calculations because these types of calculations are variational and multiple variables are allowed to change while multiple constraints are being applied.

To simplify the calculations, we choose level $k = 5$ with $l = 1$ and $r = 1$. Thus, Eq. 6 predicts $\tilde{\lambda} = 1/512$. As seen in Figure S1, if we substitute $\tilde{\lambda} = 1/512$ into $l/(n^k r^4) + \tilde{\lambda} n^k r^2 l$ for our choice

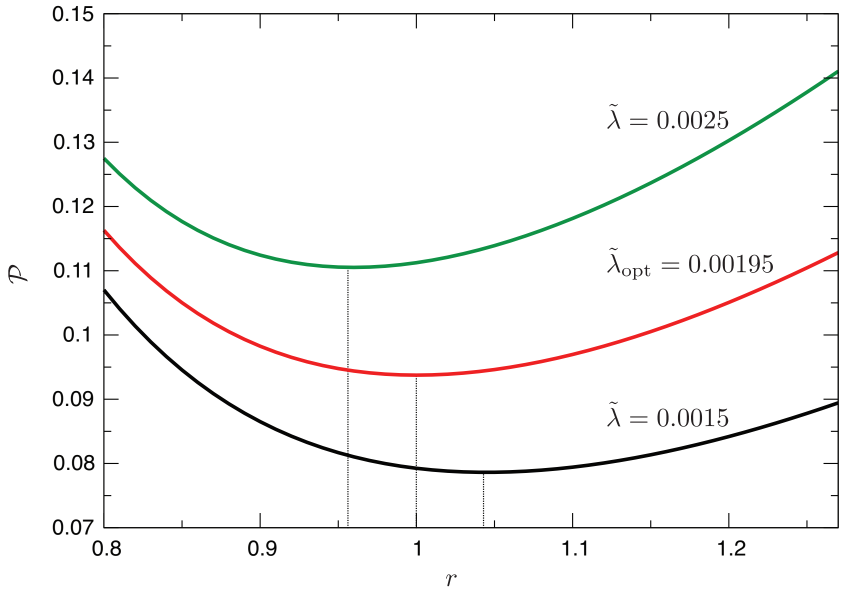

Figure S1: **Minimizing energy loss to dissipation.** Plot for an arbitrary level of the cardiovascular network $(l/(n^k r^4) + \tilde{\lambda} n^k r^2 l)$ taken from the first two terms of the objective function $\mathcal{P}$ in Eq. 4 versus the the radius of the vessel, r. For simplicity, we chose the parameters $l = 1$, $r = 1$, and $k = 5$. Using these parameters, the predicted value for the Lagrange multiplier is $\tilde{\lambda} = 2/n^{2k} r^6 = 1/512 \approx 0.00195$. We plot the first two terms of the objective function versus r for the choices of the Lagrange multiplier $\tilde{\lambda}$ at $1/512 \approx 0.00195$ (red curve) and show that the minimum does indeed occur at the chosen value of $r = 1$. We also show results for the same choice of parameters as before but with $\tilde{\lambda}$ chosen to be 0.0015 (black curve) and 0.0025 (green curve) respectively. These other values for $\tilde{\lambda}$ do not exhibit a minimum at $r = 1$, indicating that either they are not evolutionarily optimized or that the objective function constructed by WBE is incorrect.

of parameters and plot the function versus r, the minimum does indeed occur at our chosen value of $r = 1$ as it should. We also present curves $l_k/(n^k r_k^4) + \tilde{\lambda} n^k r_k^2 l_k$ with $\tilde{\lambda} = 0.0015$ and $\tilde{\lambda} = 0.0025$. If we consider only networks with fixed values of length ($l = 1$), radius ($r = 1$), level ($k = 5$), and total blood volume ($n^5 \pi$) at each level, then these other choices for $\tilde{\lambda}$ must be interpreted as cases in which either the network of the organism has not been optimized by selection or in which there are additional terms that need to be included in $\mathcal{P}$ in order to correctly optimize the network. The former corresponds to a lack of evolutionary optimization, while the latter is equivalent to the claim that the objective function constructed by WBE was incorrect and possibly lacked additional constraints. In either case different choices of $\tilde{\lambda}$ will dictate different network architectures such that the scaling ratio for the radii, $\beta_>$, varies with n to a different power or more likely is no longer simply related to n or even a scale-free ratio. Apart from vertically shifting the overall curve, we see that changes in $\tilde{\lambda}$ affect whether our network with a radius of 1 at level 5 is optimized. In Figure S1 changes in $\tilde{\lambda}$ of about 25% translate into changes in the optimal radius of about 5% and changes in the objective function of about 1%, which is a very slight amount. However, this figure is only meant to serve as a qualitative guide, and actual interpretations of selection strength or evolutionary pressure will require a much more detailed analysis that is more firmly grounded in organismal variation in network architecture and

hydrodynamics.

Finally, optimizing with respect to l_k,

$$\frac{\partial \mathcal{P}}{\partial l_k} = \frac{1}{n^k r_k^4} + \tilde{\lambda} n^k r_k^2 + 3\tilde{\lambda}_k n^k l_k^2 = 0, \tag{8}$$

yields the corresponding Lagrange multiplier as

$$\tilde{\lambda}_k = -\frac{1}{n^{2k} r_k^4 l_k^2}. \tag{9}$$

$\tilde{\lambda}_k$ represents the geometry of service volumes at level k. The meaning of space filling - see Assumption 5 in the main text - requires $\tilde{\lambda}_k$ to be the same across all levels k. To demand that $n^{2k} r_k^4 l_k^2$ be constant is equivalent - using equation (7) - to

$$\gamma = \frac{l_{k+1}}{l_k} = n^{-1/3}, \tag{10}$$

consistent with our earlier derivation of γ in Assumption 5 [1, 2].

Proportionality of blood volume and body mass

We next use the objective function $\mathcal{P}$ to relate network (blood) volume to body mass. Note that substituting equation (9) into the space filling term - the last term in equation (4) - gives $\sum_{k=0}^{N} \tilde{\lambda}_k n^k l_k^3 = -\sum_{k=0}^{N} \frac{l_k}{n^k r_k^4}$. This expression, cancels with the first term of $\mathcal{P}$, which represents the total network impedance Z_{net}. Returning to equation (1) in whole-organism notation, we are left with

$$\mathcal{P} = \lambda V_{blood} + \lambda_M M. \tag{11}$$

Now, varying the body mass of the organism and minimizing $\mathcal{P}$ as

$$\frac{\partial \mathcal{P}}{\partial M} = \lambda \frac{\partial V_{blood}}{\partial M} + \lambda_M = 0, \tag{12}$$

we find

$$\frac{\partial V_{blood}}{\partial M} = -\frac{\lambda_M}{\lambda}. \tag{13}$$

Integrating this equation yields $V_{blood} = -(\lambda_M/\lambda)M + C_0$. Demanding that $V_{blood} = 0$ for $M = 0$ gives $C_0 = 0$, and thus [1, 2]:

$$V_{blood} \propto M. \tag{14}$$

This result is crucial within the theory for comparing organisms of different sizes. As detailed in section 2.2 of the main text, the self-similar network structure provides a function mapping the number of capillaries to network volume - which is effectively V_{blood} - and V_{blood} is proportional to body mass M. At the same time, the number of capillaries in a network determines metabolic rate, as the capillaries are the only delivery channels (by Assumption 8, and their geometry is constant across organisms, by Assumption 7). Clearly, equation (14) therefore underlies nearly

every result in WBE and our main text. It is important to empasize that this calculation only derives the proportionality between V_{blood} and M for the case of dissipation minimization, not for impedance matching. It is also important to realize that the ordering of optimizations is important. First we determine the network architecture for organisms of fixed size, and then we use the results to derive the proportionality of network volume to organism size or body mass.

Minimization of energy loss to wave reflections

In larger vessels, for example near the heart, dissipation is less important, and reflection of pumped blood at vessel branch points becomes the dominant form of energy loss. In contrast to dissipation, which always causes some energy loss, reflections (and thus any energy loss attributable to them) can be completely eliminated using particular network architectures.

To derive the correct architecture for eliminating reflections, we perform a standard impedance matching calculation, identical to calculations done to minimize reflections for audio systems or electric power lines [4–7]. There are three components to pulsatile blood flow:

(a) the incident flow (labelled i) from a vessel at level k,

(b) the transmitted wave (labelled t) into a vessel at level $k+1$, and

(c) the reflected flow (labelled r) back into a vessel at level k.

The pressures, impedances, and volume flow rates for each of these components can be related to one another using the hydrodynamic version of Ohm's law

$$\begin{aligned} p_k^i &= |\dot{Q}_k^i| Z_k \\ p_k^t &= |\dot{Q}_{k+1}^t| Z_{k+1} \\ p_k^r &= -|\dot{Q}_k^r| Z_k. \end{aligned} \tag{15}$$

By continuity, the total pressure across a branching junction cannot change. Since pressure is a local (per area) quantity, we have

$$p_k^i + p_k^r = p_k^t, \tag{16}$$

independent of the number of vessels the flow divides into. Likewise, the volume flow rates must be equal at the branching junction. However, volume flow rate is a bulk quantity and thus depends on the branching:

$$|\dot{Q}_k^i| - |\dot{Q}_k^r| = n|\dot{Q}_k^t|, \tag{17}$$

where n is the branching ratio. Inserting equation (15) into (17) yields

$$p_k^i - p_k^r = \frac{nZ_k}{Z_{k+1}} p_k^t. \tag{18}$$

Adding equations (16) and (18) gives

$$2p_k^i = (1 + \frac{nZ_k}{Z_{k+1}}) p_k^t, \tag{19}$$

while subtracting them yields

$$2p_k^r = (1 - \frac{nZ_k}{Z_{k+1}})p_k^t = 2\frac{1 - \frac{nZ_k}{Z_{k+1}}}{1 + \frac{nZ_k}{Z_{k+1}}}p_k^i. \tag{20}$$

Classic impedance matching asserts that the impedance of a vessel at level k matches the impedance of the n parallel daughter vessels at level $k+1$ when

$$\frac{1}{Z_k} = \frac{n}{Z_{k+1}} \quad \text{or} \quad \frac{nZ_k}{Z_{k+1}} = 1. \tag{21}$$

When equation (21) is fulfilled, it follows from equation (20) that the volume flow rate and pressure of the reflected wave is zero, i.e., $p_k^r = 0$. Consistent with this, we see from equation (19) that the pressure of the transmitted wave exactly equals the pressure of the incident wave, $p_k^i = p_k^t$. Impedance matching, as expressed by equation (21), therefore completely eliminates reflected flow and any resultant energy loss. But what are the consequences of impedance matching for the network architecture?

For large vessels near the heart, blood flow is pulsatile and the impedance is given by

$$Z_k = c_0^2\rho/\pi r_k^2 c, \tag{22}$$

where c_0 is the Korteweg-Moens velocity, ρ is the density of blood, and c is the wave velocity [4–7]. When requiring continuity of wave properties, thus allowing only r_k to vary across levels, impedance matching as expressed by equation (21) yields $nr_{k+1}^2/r_k^2 = 1$ or the WBE result [1, 2]

$$\beta_< = \frac{r_{k+1}}{r_k} = n^{-1/2}. \tag{23}$$

This scaling ratio for radii means that the sum of the cross sectional areas of vessels at level k equals that of vessels at level $k+1$. Indeed, using equation (23), we find preservation of area, $A_{k,tot} = n^k\pi r_k^2 = n^{k+1}\pi r_{k+1}^2 = A_{k+1,tot}$. In this region near the heart, the blood does not slow down, maintaining the same velocity at which it was pumped from the heart.

As for the case of dissipation, we can investigate how strongly deviations from optimality will affect the results and begin to consider the strength of evolutionary pressures on network architecture. Based on equation (20), we define the standard reflection coefficient, R, such that $|R|^2 = |(1 - (nZ_k/Z_{k+1}))/(1 + (nZ_k/Z_{k+1}))|^2$ [4–7]. Note that when impedances are matched (equation (21)), the reflection coefficient is zero. In Figure S2 we plot the square of the reflection coefficient versus the scaling ratio for the radii $\beta = r_{k+1}/r_k$. A minimum at the value of $\beta = 0.707 \approx 2^{-1/2}$, corresponding to area-preserving branching and impedance matching, is clearly evident, and values for $|R|^2$ increase quickly away from this value for β. Indeed, comparing Figure S2 with Figure S1 naively suggests that evolutionary pressure to minimize energy loss due to reflections is greater than pressure to minimize energy loss to dissipation. This is an intriguing area for further research.

Interestingly, impedance matching for small vessels with Poiseuille flow, and thus impedance given by equation (2), leads to equation (10), recovering the same result obtained with Lagrange

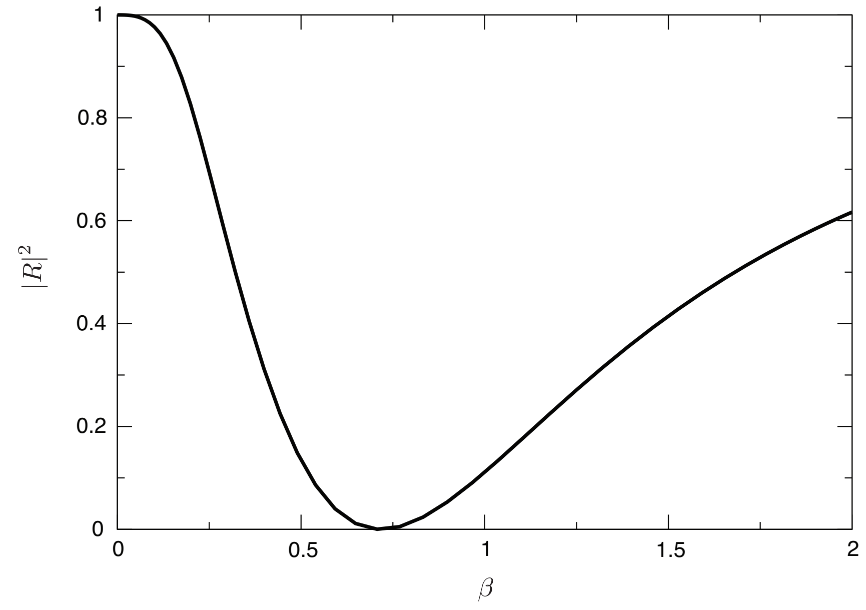

Figure S2: **Minimizing energy loss to reflection.** Plot of the reflection coefficient squared ($|R|^2 = |(1-nZ_k/Z_{k+1})/(1+nZ_k/Z_{k+1})|^2$) versus the ratio of vessel radii $\beta = r_{k+1}/r_k$ at a branching junction. The impedances are defined as in equations 34 and 35 in the supplementary material. The kinematic viscosity, the ratio of blood viscosity to blood density, is $2.57x10^{-6}s/m^2$. We choose a bifurcating branching ratio $n = 2$, and the wave frequency and the radius of the parent vessel are taken to be 1 Hz and 1.5 cm, respectively, to approximate the values for the human aorta. As long as the radius of the parent vessel is large as defined by equation 33, different choices for the wave frequency and radius of the parent vessel will change the exact values in the plot but not the shape of the curve. The plot reveals that the reflection coefficient is zero at $\beta = 0.707 \approx 2^{-1/2}$, which exactly corresponds to area-preserving branching and impedance matching.

multipliers in the case of minimizing energy loss from dissipation. Consequently, vessels whose radii scale according to the ratio (10) simultaneously minimize energy loss to both dissipation and reflections in Poiseuille flow.

Matching impedance at all levels

Section 1 and 2 were about the limiting cases of small and large vessels. In this section, we outline work originally done by Womersley for calculating the impedance to blood flow through an elastic vessel [8, 9]. This approach allows us to derive a general expression for impedance that reduces to equation (2) for small vessels, to equation (22) for large vessels, and to more complicated forms for vessels of intermediate size [1, 2]. In this way, we can calculate impedance at all levels, and study more carefully the transition from levels with mainly impedance matching to levels with mainly dissipation minimization.

As with all hydrodynamic calculations, the natural starting point are the Navier-Stokes equations [4–10]. Here we assume that non-linear turbulent flow is negligible to first order. The

general equation for blood flow then is

$$\rho \frac{\partial \mathbf{v}}{\partial t} = \mu \nabla^2 \mathbf{v} - \nabla \mathbf{p}, \tag{24}$$

where ρ denotes the density of blood, μ the viscosity, the vector $\mathbf{v}$ is the local fluid velocity at time t, and $\mathbf{p}$ is the local pressure. A similar equation, known as the Navier equation, describes the motion of vessel walls

$$\rho_w \frac{\partial^2 \mathbf{X}}{\partial^2 t} = E \nabla^2 \mathbf{X} - \nabla \mathbf{p}, \tag{25}$$

where ρ_w denotes the density of the vessel wall, E denotes the modulus of elasticity of the vessel wall, and the vector $\mathbf{X}$ is the local displacement of the wall. Since $\mathbf{X}$ is a position, its second time derivative in equation (25) is an acceleration. Likewise, the first time derivative of the fluid velocity on the left side of equation (24) is also an acceleration. Both equations (24) and (25) are coupled at the boundary of blood and vessel-wall, where the motion of one is exactly tied to the motion of the other. The components of velocity and force of fluid and vessel wall must be continuous and therefore match at the boundary. Further conditions are imposed on the outer part of the vessel wall, which is in contact with body tissue. The outer vessel wall is assumed to be nearly stationary, because it is secured by the surrounding tissue.

To solve these equations, we make a few simplifying assumptions. Blood is, to an excellent approximation, an incompressible fluid. By local conservation of fluid, we have $\nabla \cdot \mathbf{v} = 0$. Taking the vector derivative, ∇ of equation (24) thus yields the further condition that

$$\nabla^2 \mathbf{p} = 0, \tag{26}$$

which can be used to further simplify equations (24) and (25). Finally, the thickness of the vessel wall h, which appears in the equations when taking into account the boundary conditions, is small compared to the static equilibrium value of the vessel radius R, allowing further simplifying expansions. We model the heart beat as delivering an oscillatory pressure gradient, represented by a plane wave:

$$\frac{\partial \mathbf{p}}{\partial z} = p_0 e^{i\omega_0 t}, \tag{27}$$

where z is the axial direction along the vessel, ω_0 is the frequency of the wave, and p_0 is a normalization constant. All of these approximations are well grounded in the biology of the circulatory system, and deviations arising from them should be small.

Instead of carrying out the general calculations - whose result we provide below - we just highlight how the calculation is done for the simpler case of a rigid tube. Using the above approximations and boundary conditions, Fourier transforms or separation of variables can be used to analytically solve equations (24) and (25) to obtain the velocity profile in the axial direction:

$$u(r,t) = e^{i\omega_0 t} \frac{i p_0}{\rho \omega_0} \left(1 - \frac{J_0(k_0 r)}{J_0(k_0 R)} \right). \tag{28}$$

Equation (28) describes how the axial blood velocity varies with the distance from the radial center of the vessel, r, while oscillating in time t. In this expression, R is the radius or distance from the center of the vessel to the inner vessel wall, $k_0 = \sqrt{\omega_0 \rho / i\mu}$, and the function J_0 is the

zeroth-order Bessel function, which commonly occurs in problems with radial symmetry [11]. Note that at the vessel wall, $r = R$, the axial velocity is zero, while at the vessel center, $r = 0$, the axial velocity is maximal $e^{i\omega_0 t} ip_0/\rho\omega_0$.

For this rigid tube calculation, it is now straightforward to derive the volume flow rate $\dot{Q}$, because it is just the velocity profile integrated over an area element pointing in the axial direction, describing how fast each area element of fluid is flowing forward:

$$\dot{Q} = \frac{d(\text{Blood Volume})}{dt} = \int_0^R u(r,t)dA = 2\pi \int_0^R u(r,t)rdr. \tag{29}$$

Using standard identities for Bessel functions and their integrals [11, 12], this can be manipulated to yield

$$\dot{Q} = -i\frac{\pi p_0 R^4}{\mu}\frac{e^{i\omega_0 t} J_2(i^{3/2}\alpha)}{\alpha^2 J_0(i^{3/2}\alpha)}, \tag{30}$$

where $\alpha = \sqrt{\omega_0\rho/\mu}R = k_0 R/\sqrt{-i}$ is the Womersley number and J_2 is the Bessel function of second order [8, 9]. As first noted by Womersley, the character of the wave, and thus of the volume flow rate, depends crucially on α, in particular whether α is greater than or less than 1. Within human circulatory systems, the value of α is near 15 in the aorta, 5 in the arteries, 0.04 in the arterioles, and 0.005 in the capillaries [1, 2]. Using the known asymptotic expansions for Bessel functions [11, 12], one obtains that for small values of $|\alpha|$ (corresponding to vessels with small radii) the Bessel functions are power series, while for large values of $|\alpha|$ (corresponding to vessels with large radii) the Bessel functions exhibit oscillatory behavior. Consequently, equation (30) can be greatly simplified in these limits to yield

$$\begin{aligned} \dot{Q} &= \frac{\pi p_0 R^4}{8\mu} e^{i\omega_0 t} \quad |\alpha| \ll 1 \quad \text{or} \quad R \ll \sqrt{\frac{\mu}{\omega_0\rho}} \\ \dot{Q} &= \frac{\pi p_0 R^2}{\omega_0\rho} e^{i(\omega_0 t - \pi/2)} \quad |\alpha| \gg 1 \quad \text{or} \quad R \gg \sqrt{\frac{\mu}{\omega_0\rho}}. \end{aligned} \tag{31}$$

Note that different physical quantities become relevant in the two regimes. The viscosity, μ, is relevant in the small-vessel regime ($|\alpha| \ll 1$) and the density ρ is relevant in the large-vessel regime ($|\alpha| \gg 1$). Moreover, the flow in the large vessels is phase shifted by $e^{-i\pi/2} = 90°$ compared to flow in small vessels.

To deal with the multiple forms of energy loss - dissipation and reflection - we have introduced a complex analog of the volume flow rate and pressure gradients. The definition of the complex impedance, representing the difficulty of forward flow caused by both dissipation and reflections, is the ratio of the pressure to the volume flow rate:

$$Z = \frac{\Delta p}{\dot{Q}}. \tag{32}$$

A larger impedance corresponds to larger pressures that result in smaller volume flow rates. The pressure difference along the full length of the vessel, l, is $\Delta p = \int_0^l (\partial p/\partial z)dz = \int_0^l p_0 e^{i\omega_0 t}dz = p_0 e^{i\omega_0 t} l$. Thus, combining this with equations (31) and (32),

the impedance of a small and large vessel is given by [1, 2, 4–7]:

$$
\begin{aligned}
|Z| &= \frac{8\mu l}{\pi R^4} \quad |\alpha| \ll 1 \quad \text{or} \quad R \ll \sqrt{\frac{\mu}{\omega_0 \rho}} \\
|Z| &= \frac{\rho \omega_0 l}{\pi R^2} \quad |\alpha| \gg 1 \quad \text{or} \quad R \gg \sqrt{\frac{\mu}{\omega_0 \rho}}.
\end{aligned} \tag{33}
$$

The result for small vessels is exactly equation (2), representing the impedance for smooth Poiseuille flow. For larger vessels, the impedance has the same dependence on vessel radius as equation (22) and thus impedance matching will lead to the same scaling result for radii as equation (23). However, the remaining factor of $\rho\omega_0 l/\pi$ is not quite correct because of the assumption of rigid vessel walls. Nevertheless, this calculation outlines the approach to the problem and leads to the correct radial dependencies in both limits.

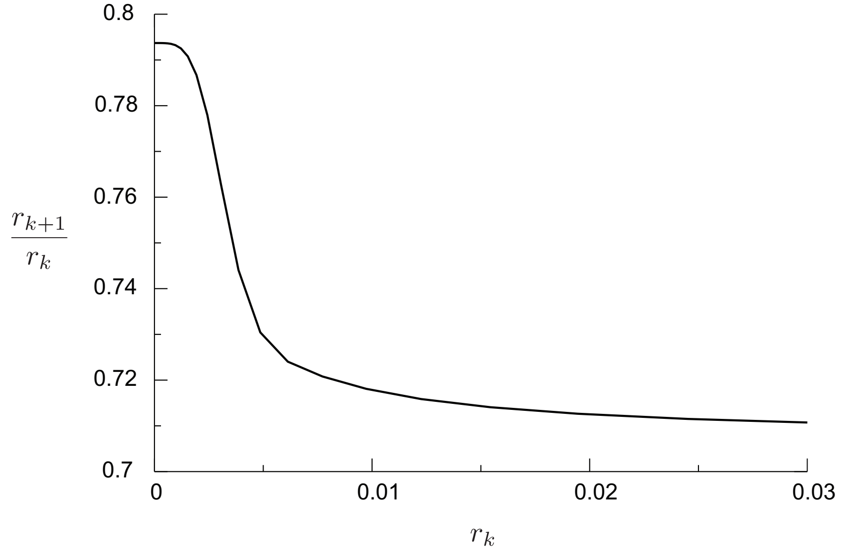

Figure S3: **Minimizing total power loss.** The graph depicts the $\beta_k \equiv r_{k+1}/r_k$ that minimizes the total power lost in going from one branching level to the next as a function of the vessel radius, r_k, in units of meters. We have chosen $n = 2$, the minimum radius to be the average value for a capillary, $r_{cap} = 8\mu m$, and the physical values for kinematic viscosity of $\mu/\rho = 2.57x10^{-6}s/m^2$ and for wave frequency of $\omega_0 = 1.17s^{-1}$. For smaller values of r_k, the minimum occurs around $\beta_k = 0.794$, and for larger values of r_k, the minimum occurs around $\beta_k = 0.710$. These values match the predicted values from WBE of $\beta_k = n^{-1/3} = 2^{-1/3} = 0.794$ and $\beta_k = n^{-1/2} = 2^{-1/2} = 0.707$ extremely well. Also, note that the transition from area-preserving to area-increasing branching begins at $r_k \approx$ 1cm and is completed by $r_k \approx$ 1mm.

Returning to the case of elastic vessel walls, the result for the impedance is

$$
Z = \frac{c_0^2 \rho}{\pi R^2 c}, \tag{34}
$$

where

$$
\left(\frac{c}{c_0}\right)^2 = -\frac{J_2(i^{3/2}\alpha)}{J_0(i^{3/2}\alpha)}, \tag{35}
$$

with c the wave velocity and c_0 the classical Korteweg-Moens velocity [1, 2, 4–7]. This expression can be shown to reduce to equations (2) and (22) in the limit of small and large vessels. Outside these limiting cases, expression (35) allows computation of the impedance for vessels of intermediate size ($\alpha \approx 1$ or $R \approx \sqrt{\frac{\mu}{\omega_0 \rho}}$), which can be used to study how the transition from pure impedance matching to pure dissipation minimization, and thus from equation (7) to equation (23), actually occurs (see Figure S3).

Accounting for the log-normal distribution in species abundance with body mass

There are many more small mammals than large ones. This bias in the body mass distribution is reflected, likely even accentuated, in available empirical data for mammalian metabolic rate and body size. Studies have shown that the distribution of body masses across species is approximately log-normal [13–15]. The shape of this distribution would be inconsequential if the metabolic rate to body mass relationship were a perfect power law with no scatter. As demonstrated above, however, the WBE model does not predict a pure power law, and as such the distribution of organism sizes could have an influence on the measured value of the exponent. Furthermore, different species of the same average body mass exhibit scatter in their average basal metabolic rates, with measurement inaccuracies further compounding the observed scatter. It stands to reason that the more the body size distribution is skewed towards small mammals, the more it will emphasize finite-size effects, an issue we shall revisit in section 2.3. At the same time, this bias also amplifies the data scatter for small mammals, washing out their influence on the overall fit.

One way to account for the effects of the log-normal body mass distribution on our analysis of the scaling exponent in section 2.1 is to logarithmically bin the artificial data such that bin-averages are evenly distributed along the abscissa of our plots [16]. Another way is to sample networks directly from a log-normal size distribution, and then perform linear regressions on that data. We shall do the latter.

To proceed, we first need to determine the parameters of the appropriate log-normal distribution. The number of levels in the cardiovascular system scales approximately logarithmically with body mass [1]. Consequently, a log-normal distribution for body mass corresponds to a normal distribution in the number of levels. Since the number of levels, N, is just the logarithm of body mass, $\ln M$, scaled by a constant factor that depends on the branching ratio [1], there is an approximate equivalence between Z-scores for the $\ln M$ and the N distributions.

In order to generate a distribution from which to sample model organisms, we must determine an average and a standard deviation for the N-distribution. To do this, we exploit the Z-score equivalence. According to WBE, the smallest mammal (a shrew) has 25 levels assuming $n = 2$. Yet, the smallest organism included in the empirical data collected by Savage *et al.* [16] has a body mass about 5 times larger than a shrew. We therefore take the smallest organism in this analysis to have 27 levels since the blood volume of an organism with 27 levels is $\sim$5 times the blood volume of an organism with 25 levels in our calculations. The largest organism in the Savage data set is about 1.5×10^6 the size of the smallest organism, allowing us to determine a reasonable

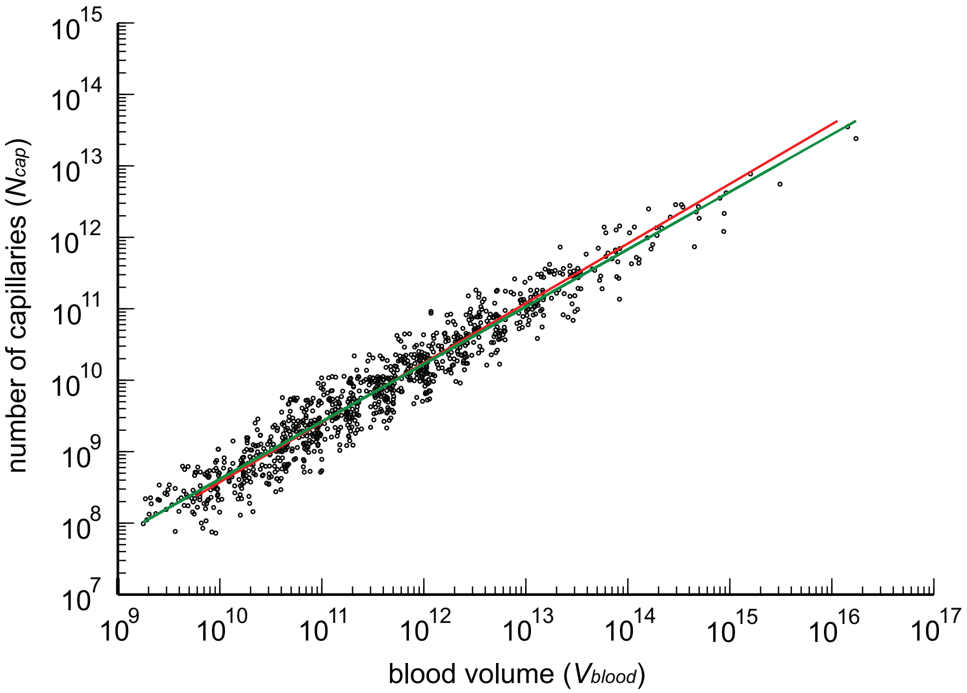

Figure S4: **Log-normal sampling bias for small networks.** A single realization of 1000 numerically generated data points for networks with a branching ratio of $n = 2$ built with area-preserving branching for large vessels and area-increasing branching for small vessels. The transition between these regimes is always $\bar{N} = 24$. The networks are generated by sampling the number of levels from a log-normal distribution. (See text for details.) Based on empirical data [16], the underlying distribution ranges from a minimum of 27 levels to a maximum of 44 levels with an average of 32 and a standard deviation of 4. The scatter is generated by multiplying the blood volume and number of capillaries by two random numbers drawn from a uniform distribution on the interval $[0.3, 1.7]$. This procedure generates one order of magnitude scatter in metabolic rate for a given mass, mimicking the variation observed in empirical data for mammals. Red line: Fit to artificial data without scatter - exponent is 0.83. Green line: Fit to artificial data with added scatter - exponent is 0.8.

maximal N. Equating the Z-scores for the $\ln M$ distribution and the N distribution, we obtain

$$\begin{aligned} Z_S &= \frac{\ln M_S - \langle \ln M \rangle}{\sigma_{\ln M}} = \frac{N_S - \langle N \rangle}{\sigma_N} \\ Z_L &= \frac{\ln M_L - \langle \ln M \rangle}{\sigma_{\ln M}} = \frac{N_L - \langle N \rangle}{\sigma_N}, \end{aligned} \tag{36}$$

where Z_S and Z_L are the relevant Z-scores for M_S and M_L (the smallest and largest body masses in the data), σ is the standard deviation of the variable identified in the subscript, and $\langle \cdot \rangle$ denotes an average. Since Z_S and Z_L can be determined from empirical data, we are left with two equations and two unknowns. From this we determined the values of $\langle N \rangle$ and σ_N to be 32 and 4, respectively. These parameters define a Gaussian distribution from which we sampled 1000 values for N. With these values we generated corresponding WBE networks (with $n = 2$ and $\bar{N} = 24$),

obtaining a sample biased towards small networks, much like in the empirical data set. From this computational data set we determined a scaling exponent of 0.83, which is even larger than the exponent observed when masses are sampled uniformly, apparently compounding the discrepancy between the finite-size WBE model and empirical data.

One might object that our procedure ignores measurement error, differences between species of the same average body mass, and other sources of scatter in the data, since each artificial network with the same number of levels has identical values for N_{cap} and V_{blood}. In order to model the scatter observed in empirical data, we randomized the log-normally distributed simulated data. For each (V_{blood}, N_{cap}) pair, we chose two random numbers, q_1 and q_2, from a uniform distribution on the interval $[0.3, 1.7]$ and replaced (V_{blood}, N_{cap}) with ($q_1 V_{blood}$, $q_2 N_{cap}$). This procedure creates a scatter of about one order of magnitude in metabolic rate for each body mass value along the abscissa, similar to the scatter observed in the empirical data of Savage *et al.* [16]. Figure S4 displays a single realization of our artificial log-normally distributed data with scatter. The addition of scatter in this case reduced the exponent from 0.83 to 0.80 - a step in the right direction, but still not in satisfying agreement with empirical data. Applying this procedure to sets of unscattered data with scaling exponents close to 3/4 or data with a larger degree of scatter (which washes out the log-normal bias in the overall fit) may lower the exponent to less than 3/4. We conclude that accounting for a log-normal distribution of body-mass does not improve the predictions of the WBE model.

Impact of finite-size corrections on additional WBE predictions

In addition to the 3/4 exponent for metabolic rate (in the limit of infinite network size), the WBE model predicts the scaling of many organismal rates, times, and lengths. Table 1 of the original WBE paper [1] provides a sample of such predictions. In this section we recalculate that table in the light of finite-size effects. We have already predicted in the main text how these effects impact the scaling of capillary number with blood volume and thus body mass. In most cases, organismal rates, times, or lengths can be related to the number of capillaries, and directly inherit their finite-size scaling corrections. The variables listed in column 1 are the same as in WBE. The predicted scaling exponents in column 2 report our finite-size corrections based on the canonical WBE model. Column 3 reports the observed scaling exponents as reported in the original Table 1 of WBE [1]. Column 4 shows the asymptotic results reported in the original WBE paper [1].

In some cases the asymptotic and finite-size predictions agree; in other cases, they don't. For circulation time, WBE report just the scaling exponent of 1/4 that holds for mammals in the asymptotic limit of infinite size. However, for small mammals the circulation time should actually have a scaling exponent close to 0. This transition in the scaling exponent is exactly due to the transition from area-preserving to area-increasing branching in the original WBE model. The theory would gain strong support if a transition or this type of curvature could be observed in empirical data for circulation times. WBE also report a scaling exponent of $-1/12$ for the density of capillaries. We are unable to re-create this original prediction. Indeed, we believe the correct prediction should be $-1/4$.

The derivation of these scaling exponents is left unexplained in the original WBE paper. As an

Variable	Finite-Size Scaling	Observed	WBE Scaling
Aorta radius r_0	$0.81/2 = 0.405$	0.36	$(3/4)/2 = 0.375$
Aorta pressure Δp_0	0	0.032	0
Aorta blood velocity u_0	0	0.07	0
Blood volume V_b	1	1.00	1
Circulation time (large)	$0.81/3 = 0.27$	0.25	$(3/4)/3 = 0.25$
Circulation time (small)	0	ND	0 (unreported)
Circulation distance l	$0.81/3 = 0.27$	ND	$(3/4)/3 = 0.25$
Cardiac stroke volume	$4 * 0.81/3 = 1.08$	1.03	$(4/3) * (3/4) = 1$
Cardiac frequency ω	$-0.81/3 = -0.27$	-0.25	$-(3/4)/3 = -0.25$
Cardiac output $\dot{Q}_0$	0.81	0.74	$3/4 = 0.75$
Number of capillaries N_{cap}	0.81	ND	$3/4 = 0.75$
Service volume radius	$(0.81 - 1)/3 = -0.0633$	ND	$(3/4 - 1)/3 = 0.083$
Womersley number α	$0.81/3 = 0.27$	0.25	$(3/4)/3 = 0.25$
Density of capillaries	$0.81 - 1 = -0.19$	-0.095	$-1/12 = -0.083$ *
O_2 affinity of blood	$(0.81 - 1)/3 = -0.0633$	-0.089	$(3/4 - 1)/3 = -0.083$
Total resistance Z	-0.81	-076	$-3/4 = -0.75$
Metabolic rate B	0.81	0.74	$3/4 = 0.75$

Table S1: The table reports our finite-size corrections to the predictions listed in Table 1 of WBE [1]. Predictions refer to the exponent that governs the scaling relation between the variable in column 1 and body mass M. This table should be read as a further evaluation of the WBE model for finite vascular networks. * We cannot reproduce the WBE derivation for the capillary density. See text for details.

additional service to the community, we use this Supplementary Material as a venue to summarize the origins of these scaling relations. Many of our derivations will be expressed in terms of N_{cap}; using our finding that $N_{cap} \propto M^{0.81}$, these results can be easily converted into scaling predictions as they relate to body mass M. This is how they are reported in our Table 1.

Aorta radius r_0: The aorta radius can be related to capillary radius and number by using the scaling relations $\beta_<$ and $\beta_>$ (equations (3-4) in the main text) to obtain $r_0 \propto N_{cap}^{1/2} n^{\bar{N}/6} r_{cap}$. Since r_{cap}, n, and $\bar{N}$ (the number of area-increasing levels in the network) are all invariant with respect to body mass M, we have that $r_0 \propto N_{cap}^{1/2} \propto M^{-0.405}$. In this last step we used our result that $N_{cap} \propto V_{blood}^{0.81} \propto M^{0.81}$ when finite-size corrections are included.

Aorta pressure Δp_0: The aorta pressure scales with an exponent of 0 because $\Delta p_0 = \dot{Q}_0 Z$ and $\dot{Q}_0$ and Z scale inversely to one another.

Aorta blood velocity u_0: The aorta blood velocity times the cross-sectional area of the aorta is just the volume flow rate of blood $\dot{Q}_0 = u_0 A_0$. Since $\dot{Q}_0 \propto B \propto N_{cap}$ (by assumption) and $A_0 \propto r_0^2 \propto N_{cap}$ (from our previous arguments), their scaling cancels out, leaving u_0 as an invariant with respect to body mass.

Blood volume V_b: Results from the Lagrange multiplier calculations in earlier sections of the Supplementary Material show that blood volume is directly proportional to body mass,

$V_b \propto M$.

Circulation time: The circulation time is the overall time it takes the blood to traverse each of the k levels: $\sum_{k=0}^{N} l_k/u_k$. For area-increasing or dissipative levels, there are always $\bar{N} = N - \bar{k}$ levels; l_k and u_k are both determined by the invariant length and flow rate of a capillary and distance (in terms of levels) from the capillary: $l_k = n^{(N-k)/3} l_{cap}$ by the scaling ratio γ, and $u_k = n^{(N-k)/3} u_{cap}$ by conservation of fluid (i.e., $\dot{Q}_k = \dot{Q}_{k+1}$). Thus, the time to traverse a vessel at level k is $l_k/u_k = l_{cap}/u_{cap}$, which is independent of k, and the circulation time for the smallest mammal is just $\sum_0^N l_{cap}/u_{cap} = \bar{N}(l_{cap}/u_{cap})$. Including area-preserving branching will add time to this constant value. For small mammals, this additional time will be negligible, resulting in a scaling exponent of 0. For large mammals, area-preserving branching along with conservation of fluid demands that $u_k = u_0 \propto M^0$ from our earlier arguments. When the time to flow through the area-preserving regime begins to dominate, the circulation time for large mammals scales as $\sum_0^{\bar{k}} l_k = \sum_0^{\bar{k}} n^{(N-k)/3} l_{cap} \propto N_{cap}^{1/3}$, where we used the scaling ratio γ to relate l_k to l_{cap}.

Circulation distance l: Circulation distance is just the sum over the lengths of the vessels, and thus scales as $N_{cap}^{1/3}$.

Cardiac stroke volume: The cardiac stroke volume is assumed to equal the volume of the aorta, $V_0 \propto r_0^2 l_0$. We have previously derived $r_0 \propto N_{cap}^{1/2}$; using the scaling ratio γ, it is straightforward to show that $l_0 \propto N_{cap}^{1/3}$, hence $V_0 \propto N_{cap}^{4/3}$.

Cardiac frequency ω: The product of the cardiac stroke volume V_0 and the cardiac frequency ω is equal to the volume flow rate $\omega V_0 = \dot{Q}_0$; using our prior derivations, we have $\omega \propto N_{cap}^{-1/3}$. This result may be surprising, because cardiac frequency or heart rate is typically assumed to scale as $\omega \propto B/M$. Heart volume is typically assumed to scale linearly with body mass and metabolic rate is assumed to scale as the volume flow rate of the aorta, so that $\omega = \dot{Q}_0/V_0 \propto B/M$. Indeed, most biological times are assumed to scale as B/M [16–18] . However, within the strict interpretation of the WBE model, an increase in metabolic rate is associated with an increase in the number of capillaries, which is, in turn, associated with an increase in heart volume because of the relationships between aorta length and radius on the one hand and the number of capillaries on the other hand. This increase in heart volume allows for more blood to be pumped with each contraction of the heart, and consequently, for a lower heart rate or cardiac frequency to supply the required amount of blood. Intriguingly, when the scaling for metabolic rate is $B \propto N_{cap} \propto M^{3/4}$, we obtain the same result along both arguments: $\omega \propto N_{cap}^{-1/3} \propto M^{-1/4}$ and $\omega \propto B/M \propto M^{-1/4}$. Any value for the scaling exponent other than 3/4, however, will yield different predictions for the two lines of argumentation. For example, for our finite-size corrected exponent of 0.81, the strict interpretation of the WBE model yields $\omega \propto N_{cap}^{-1/3} \propto M^{-0.27}$, while the second line of reasoning yields $\omega \propto B/M \propto N_{cap}/M \propto M^{-0.19}$.

Cardiac output $\dot{Q}_0$ & number of capillaries N_{cap}: The cardiac output or volume flow rate $\dot{Q}_0$ and N_{cap} are both directly proportional to metabolic rate, so they must also be directly proportional to one another. Following our results in the main text, the number of

capillaries N_{cap} scales as $V_{blood}^{0.81} \propto M^{0.81}$ and combines with the above arguments to yield scaling exponents for each variable with body mass, M, as reported in Table 1.

Service volume radius: WBE argue that each capillary feeds a collection of cells known as a service volume, V_S. Together, these volumes add up to the volume of the entire body, V_{body}. Hence, $N_{cap}V_S = V_{body}$, and the service volume radius scales as $r_S \propto (V_S)^{1/3} \propto (V_{body}/N_{cap})^{1/3} \propto M^{(1-0.81)/3}$.

Womersley number α: The Womersley number is defined to be $\alpha = \sqrt{\omega\rho/\mu} r$. Noting that the density ρ and viscosity μ are constants and using the previously derived scaling relations for the cardiac frequency ω and the aorta radius r_0 gives the predicted scaling for α.

Density of capillaries: The density of capillaries is N_{cap}/V_{body}. Assuming a constant density ($V_{body} \propto M$) yields $M^{(0.81-1)}$.

Oxygen affinity of blood & total resistance Z: Following the arguments given explicitly in WBE [1], we derive that the oxygen affinity of blood should scale as $M^{(1-0.81)/3}$, and that the total resistance for non-pulsatile blood flow scales as $Z \propto 1/N_{cap}$.

References

1. West GB, Brown JH, Enquist BJ (1997) A general model for the origin of allometric scaling laws in biology. Science 276:122–126.
2. West GB, Brown JH (2000) Scaling in Biology. Oxford: Oxford University Press.
3. Marion JB, Thornton ST (1988) Classical Dynamics of Particles and Systems. San Diego: Harcourt Brace Jovanovich.
4. Caro CG, Pedley TJ, Schroter RC, Seed WA (1978) The Mechanics of Circulation. Oxford, UK: Oxford University Press.
5. Fung YC (1984) Biodynamics. New York: Springer Verlag.
6. Fung YC (1990) Biomechanics: Motion, Flow, Stress, and Growth. New York: Springer-Verlag.
7. Zamir M (2005) The Physics of Coronary Blood Flow. New York: Springer.
8. Womersley JR (1955) Method for the calculation of velocity, rate of flow, and viscous drag in arteries when the pressure gradient is known. J Physiol 127:553–563.
9. Womersley JR (1955) Oscillatory motion of a viscous liquid in a thin-walled elastic tube–i: The linear approximation for long waves. Philos Mag 46:199–221.
10. Landau LD, Lifshitz EM (1978) Fluid Mechanics. Oxford: Pergamon Press.
11. Abramowitz M, Stegun IE (1972) Handbook of mathematical functions with formulas, graphs and mathematical tables. Washington, DC: U.S.G.P.O.
12. Bender CM, Orszag SA (1999) Advanced Mathematical Methods for Scientists and Engineers. New York, NY: Springer.
13. Kozlowski J, Gawelczyk AT (2002) Why are speciesÕ body size distributions usually skewed to the right? Funct Ecol 16:419–432.

14. Kozlowski J, Konarzewski M (2004) Is west, brown and enquist's model of allometric scaling mathematically correct and biologically relevant? Ecology 18(2):283–289.

15. White EP, Ernest SKM, Kerkhoff AJ, Enquist BJ (2007) Relationships between body size and abundance in ecology. Trends in Ecol and Evol 22:323–330.

16. Savage VM, Gillooly JF, Woodruff WH, West GB, Allen AP, et al. (2004) The predominance of quarter-power scaling in biology. Func Ecol 18:257–282.

17. Schmidt-Nielsen K (1983) Scaling: Why Is Animal Size So Important? Cambridge: Cambridge University Press.

18. Gillooly JF, Charnov EL, West GB, Savage VM, Brown JH (2002) Effects of size and temperature on developmental time. Nature 417:70–73.

Figures

Figure S1: Minimizing energy loss to dissipation.

Plot for an arbitrary level of the cardiovascular network $(l/(n^k r^4) + \tilde{\lambda} n^k r^2 l)$ taken from the first two terms of the objective function $\mathcal{P}$ in Eq. 4 versus the the radius of the vessel, r. For simplicity, we chose the parameters $l = 1$, $r = 1$, and $k = 5$. Using these parameters, the predicted value for the Lagrange multiplier is $\tilde{\lambda} = 2/n^{2k}r^6 = 1/512 \approx 0.00195$. We plot the first two terms of the objective function versus r for the choices of the Lagrange multiplier $\tilde{\lambda}$ at $1/512 \approx 0.00195$ (red curve) and show that the minimum does indeed occur at the chosen value of $r = 1$. We also show results for the same choice of parameters as before but with $\tilde{\lambda}$ chosen to be 0.0015 (black curve) and 0.0025 (green curve) respectively. These other values for $\tilde{\lambda}$ do not exhibit a minimum at $r = 1$, indicating that either they are not evolutionarily optimized or that the objective function constructed by WBE is incorrect.

Figure S2: Minimizing energy loss to reflection.

Plot of the reflection coefficient squared $(|R|^2 = |(1 - nZ_k/Z_{k+1})/(1 + nZ_k/Z_{k+1})|^2)$ versus the ratio of vessel radii $\beta = r_{k+1}/r_k$ at a branching junction. The impedances are defined as in equations 34 and 35 in the supplementary material. The kinematic viscosity, the ratio of blood viscosity to blood density, is $2.57x10^{-6}s/m^2$. We choose a bifurcating branching ratio $n = 2$, and the wave frequency and the radius of the parent vessel are taken to be 1 Hz and 1.5 cm, respectively, to approximate the values for the human aorta. As long as the radius of the parent vessel is large as defined by equation 33, different choices for the wave frequency and radius of the parent vessel will change the exact values in the plot but not the shape of the curve. The plot reveals that the reflection coefficient is zero at $\beta = 0.707 \approx 2^{-1/2}$, which exactly corresponds to area-preserving branching and impedance matching.

Figure S3: Minimizing total power loss.

The graph depicts the $\beta_k \equiv r_{k+1}/r_k$ that minimizes the total power lost in going from one branching level to the next as a function of the vessel radius, r_k, in units of meters. We have chosen $n = 2$, the minimum radius to be the average value for a capillary, $r_{cap} = 8\mu m$, and the physical values for kinematic viscosity of $\mu/\rho = 2.57x10^{-6}s/m^2$ and for wave frequency of $\omega_0 = 1.17s^{-1}$. For smaller values of r_k, the minimum occurs around $\beta_k = 0.794$, and for larger values of r_k, the minimum occurs around $\beta_k = 0.710$. These values match the predicted values from WBE of $\beta_k = n^{-1/3} = 2^{-1/3} = 0.794$ and $\beta_k = n^{-1/2} = 2^{-1/2} = 0.707$ extremely well. Also, note that the transition from area-preserving to area-increasing branching begins at $r_k \approx$ 1cm and is completed by $r_k \approx$ 1mm.

Figure S4: Log-normal sampling bias for small networks.

A single realization of 1000 numerically generated data points for networks with a branching ratio of $n = 2$ built with area-preserving branching for large vessels and area-increasing branching for small vessels. The transition between these regimes is always $\bar{N} = 24$. The networks are generated by sampling the number of levels from a log-normal distribution. (See text for details.)

Based on empirical data [16], the underlying distribution ranges from a minimum of 27 levels to a maximum of 44 levels with an average of 32 and a standard deviation of 4. The scatter is generated by multiplying the blood volume and number of capillaries by two random numbers drawn from a uniform distribution on the interval $[0.3, 1.7]$. This procedure generates one order of magnitude scatter in metabolic rate for a given mass, mimicking the variation observed in empirical data for mammals. Red line: Fit to artificial data without scatter - exponent is 0.83. Green line: Fit to artificial data with added scatter - exponent is 0.8.
